# The international Red Cross and nurses in the context of the Brazilian press (1864–1907)*

**DOI:** 10.1590/1980-220X-REEUSP-2025-0420en

**Published:** 2026-10-26

**Authors:** Adriana Saturnino Mazziero, Luciana Barizon Luchesi, Fernando Porto, Claudia Labriola, Paulo Joaquim Pina Queirós

**Affiliations:** 1 Universidade de São Paulo, Escola de Enfermagem de Ribeirão Preto, Ribeirão Preto, SP, Brazil.; 2 Universidade Federal do Estado do Rio de Janeiro, Escola de Enfermagem Alfredo Pinto, Rio de Janeiro, RJ, Brazil.; 3 Universidade de Coimbra, Escola Superior de Enfermagem, Unidade de Investigação em Ciências da Saúde: Enfermagem, Portugal.

**Keywords:** Nursing, History of Nursing, Red Cross, Periodical, Mass Media

## Abstract

**Objective:**

To investigate the visibility of the Red Cross and the work of nurses at the national and international levels during the period 1864–1907.

**Method:**

A historical study employing documentary analysis of newspaper articles, with the Brazilian Digital Newspaper Archive serving as the primary source.

**Results:**

A total of 37 news articles were identified, 21 in the Jornal do Commercio and 16 in the Gazeta de Notícias. Internationally, most articles addressed armed conflicts, highlighting the Red Cross’s role in blood banks, emphasizing the importance of nursing in wartime and the need for professional training. Nationally, the Federalist Revolution (1893–1895) stands out, with the organization of relief commissions and the dispatch of volunteers—such as nurses, doctors, and religious figures—to carry out humanitarian work during internal conflicts and to build an identity linked to the international principles of the Red Cross.

**Conclusion:**

The Brazilian press from 1864 to 1907 contributed to the promotion of the Red Cross and the recognition of the importance of wartime nursing.

## INTRODUCTION

The Brazilian Red Cross was founded in 1908, although the idea had been circulating since the 19th century. It is mentioned in several classic works on the history of healthcare and nursing^(1,2,3,4)^. The context leading up to the institution’s emergence in Brazil remains largely unexplored. This is because, while there is a wealth of scholarly work in the form of articles, books, dissertations, and theses, most of it begins roughly two to three years before the institution’s creation, with a few exceptions^(5)^.

It was from this perspective that we set out to investigate what was happening around the world regarding the Red Cross’s activities. To this end, we turned to journalistic sources, as they are replete with reports and news stories that capture aspects of the traces and clues left by the past for the present, serving as documentation for the construction of historical narrative^(6)^. Thinking along these lines is one way to understand the past, even if only in a plausible way, since it remains there^(7)^.

It is a fact that the International Red Cross was founded in 1863 in Geneva, Switzerland, and that one of its pioneers was Henry Dunant. He witnessed the Battle of Solferino, considered one of the bloodiest and most decisive battles of the invasion of Piedmont-Sardinia (1859), which saw the victory of Napoleon III’s French troops over the Austrian military forces. This led to the publication of *Memoirs of Solferino*, which underwent title revisions and was reprinted several times. Soon, there was a growing awareness of the need to create an organization to care for the wounded and needy in times of war^(8)^.

The Red Cross initiative spread throughout the world, and trained volunteers were needed to serve on the battlefields as well as during times of disaster. This brings to mind the role of nursing on the battlefield, such as in the Crimean War (1853–1856), with Florence Nightingale, and in the War of the Triple Alliance, with Anna Justina Ferreira Nery (1864–1870)—volunteers who have brought prominence and visibility to the profession since the 19th century^(9)^.

In light of the events and arguments presented, the aim of this study was to investigate the visibility of the Red Cross and nurses in Brazilian news reports prior to the establishment of the Brazilian Red Cross.

## METHOD

### Study Type or Design

A historical study within the field of social history, utilizing documentary analysis. To this end, we argue for the application of this method based on historiographical practice^(10)^, when there is a combination of discourses and contextual elements in the production of texts based on journalistic sources. These serve as vestiges of the past, as they reflect a certain mindset that prevailed at the time regarding the investigation^(11)^.

The time frame covered extends from the founding of the International Red Cross (IRC) in 1864 to 1907, the year preceding the founding of the Brazilian Red Cross (BRC).

### Selection Criteria

The searches were conducted in the newspapers *Jornal do Commercio* and *Gazeta de Notícias*, which were chosen at random. However, we note that these publications had a significant impact on the market; in fact, *Jornal do Commercio* was often compared to *New Times* in Brazil. This is due to the innovations they introduced^(12)^, as well as for being historical documents of relevance to research in general history and nursing^(13,14)^.

### Data Collection

The search for information was conducted in the Brazilian Digital Newspaper Archive of the National Library, using the following criteria: inclusion of the term “nurse” combined with the phrase “Red Cross” within the specified time frame, excluding advertisements, promotional material, cartoons, and other similar items.

### Data Analysis and processing

To organize the data, an analysis matrix was applied with the following variables: journal title, editorial line, price per issue, journal address, journal editors, article title, publication date, publication page, article author, news summary, names of people mentioned, and location of the news context (country, state, city, or similar).

The news articles were transcribed in full using the original spelling, and their content, through data analysis, was cross-referenced to identify thematic themes^(5)^ and subsequently with the literature on adherence, for the construction of the historical narrative.

### Ethical Considerations

The ethical and legal aspects of conducting the research were governed by Resolution No. 510/2016 and Copyright Law No. 9,610/1998.

## RESULTS

The results identified 37 newspaper articles, all of which included headlines but none of which featured images: Jornal do Commercio and Gazeta de Notícias. These articles provide textual and contextual elements for the historical narrative.

The results presented in Chart 1 show that the offices were located in downtown Rio de Janeiro. Regarding prices, it is worth noting that the *Jornal do Commercio*, compared to the *Gazeta de Notícias*, was more affordable to consumers due to its lower price. To give a rough idea, during the period from 1903 to 1907, workers earned a monthly wage of approximately 78$000, and with this amount they paid rent ranging from 8$000 to 30$000^(15)^. Understanding the difference in newspaper sales allows us to argue that the Jornal do Commercio carried a greater volume of content compared to the Gazeta de Notícias. Thus, from a historical perspective, it can be explained that the Jornal do Commercio was founded in 1828, before the Gazeta de Notícias, which was established in 1875. This implies that the former newspaper had a longer commercial tradition, which may justify a lower price for the reader and a greater volume of published content.

**Chart 1:** Newspaper identification data (1864–1907) – Ribeirão Preto, SP, 2025.

Identification information	Jornal do commercio	Gazeta de notícias
Address	Rua do Ouvidor, 61, Rio de Janeiro	Rua do Ouvidor, n. 70, Rio de Janeiro – tipografia: Rua Sete de Setembro, n. 72, Rio de Janeiro – escritório
Price per copy	$30,000 (1870–1889) $36,000 (1892) $40,000 – $50,000 (1893) $60,000 (1898)	$40,000(1870–1889) $60,000 (1891) $100,000 (1893–1907)
Responsible parties	Julio Constancio de Villeneuve (1870–1879) Typografia de J. Villeneuve & C. (1880–1889) e Propriedade de Rodrigues & C. (13/01/1891)	Unidentified (1870–1879) Araujo & Mendes (1884–1907)
Editorial policy	Moderate conservative	Neutral
Number of news articles	21	16
Total number of news articles	37 news	

Another notable finding was the use of headlines that are attractive to readers, as these are what drive sales^(16)^(Chart 2). In this line of thinking, the citation of prominent figures (politicians, military personnel, nurses, doctors, among others) in the articles aims to lend credibility to the news coverage^(17)^, linked to argumentation strategies involving the application of the Attention, Interest, Desire, and Action (AIDA) technique, which leads to the formation of public opinion, especially among unsuspecting readers through the power of language^(16)^.

**Chart 2:** Distribution of newspaper articles by source, date, and title – Ribeirão Preto, SP, 2025.

Name of the newspaper	Article title Portuguese	Article title English
1. Jornal do Commercio, 05/04/1875, p.3	Sociedade de socorros aos soldados e doentes da armada e do exército. Pariz, 8 de março de 1875	Society for the Relief of Soldiers and the Sick in the Navy and Army. Paris, March 8, 1875
2. Gazeta de Noticias, 03/02/1882, p.2	A agitação Niilista	The Nihilist Uprising
3. Jornal do Commercio, 21/05/1893, p.3	Para os irmãos que sofrem	For Our Suffering Brothers
4. Gazeta de Noticias, 23/05/1893, p.2	Os Feridos	The Wounded
5. Jornal do Commercio, 28/05/1893, p.2	Convenção da Cruz Vermelha	Red Cross Convention
6. Gazeta de Noticias, 30/05/1893, p.2	Os Feridos	The Wounded
7. Jornal do Commercio, 06/06/1893, p.1	Pelos irmãos que sofrem	For our suffering brothers
8. Gazeta de Noticias, 06/06/1893, p.2	Feridos	The Wounded
9. Jornal do Commercio, 10/06/1893, p.1	Pelos irmãos que sofrem	For our suffering brothers
10. Gazeta de Noticias, 21/07/1893, p.1	Os Feridos	The Wounded
11. Gazeta de Noticias, 18/12/1895, p.2	Berlim, 2	Berlin, 2
12. Jornal do Commercio, 04/05/1896, p.3	Italianos na Africa	Italians in Africa
13. Jornal do Commercio, 21/03/1897, p.1	Telegrammas. Montevidéu, 20 de março	Telegrams. Montevideo, March 20
14. Jornal do Commercio, 24/03/1897, p.1	A Revolução do Estado Oriental	The Revolution in the Eastern State
15. Gazeta de Noticias, 02/01/1898, p.1	Washington, 1	Washington, 1
16. Gazeta de Noticias, 06/02/1898, p.1	Washington, 5	Washington, 5
17. Gazeta de Noticias, 27/04/1898, p.1	Washington, 5	Washington, 5
18. Jornal do Commercio, 06/11/1899, p.1	Pariz, 5 de novembro	Paris, November 5
19. Jornal do Commercio, 21/12/1899, p.1	O Dr. Camara Pestana	Dr. Camara Pestana
20. Gazeta de Noticias, 23/12/1899, p.3	Transvaal – Londres	Transvaal – London
21. Gazeta de Noticias, 12/07/1900, p.1	Boxers – A Cruz Vermelha– Roma, 11	Boxers – The Red Cross – Rome, 11
22. Gazeta de Noticias, 18/04/1901, p.2	Os boers em Portugal	The Boers in Portugal
23. Jornal do Commercio, 05/02/1901, p.1	Várias Noticias Washington, 5 de fevereiro	Various News Items Washington, February 5
24. Jornal do Commercio, 08/06/1901, p.2	Sociedade da Cruz Vermelha	Red Cross Society
25. Jornal do Commercio, 18/05/1901, p.2	As mulheres e a guerra	Women and War
26. Jornal do Commercio, 08/06/1901, p.2	Sociedade da Cruz Vermelha	Red Cross Society
27. Gazeta de Noticias, 08/11/1903, p.2	A vida no hospital S. Sebastião	Life at St. Sebastian Hospital
28. Jornal do Commercio, 24/02/1904, p.1	Telegrammas – Roma, 24 de fevereiro	Telegrams – Rome, February 24
29. Jornal do Commercio, 28/04/1904, p.1	A guerra Russo-Japoneza	The Russo-Japanese War
30. Jornal do Commercio, 07/07/1904, p.1	Constantinopla. 6 de julho	Constantinople. July 6
31. Jornal do Commercio, 15/09/1904, p.1	S. Petersburgo, 14	St. Petersburg, 14
32. Gazeta de Noticias, 25/09/1904, p.3	Henri Dunant	Henri Dunant
33. Jornal do Commercio, 05/10/1904, p.2	Várias Noticias	Various News
34. Jornal do Commercio, 22/02/1905, p.1	Londres, 21	London, 21
35. Gazeta de Noticias, 24/04/1906, p.2	Tokio. 23	Tokyo. 23
36. Jornal do Commercio, 24/05/1907, p.2	A crise religiosa em França (Do nosso correspondente). Pariz, 30 de Maio de 1907	The Religious Crisis in France (From our correspondent). Paris, May 30, 1907
37. Gazeta de Noticias, 14/06/1907, p.2	Inglaterra – Gazeta – Londres, 13	England – Gazette – London, 13

Source: Brazilian Digital Newspaper Archive – https://bndigital.bn.gov.br/hemeroteca-digital/.

The absence of images in the selected articles is understandable, even though the photojournalism dates back to 1842^(18)^, these were more common in weekly publications, but this did not preclude their publication in daily newspapers, despite the substantial investment required.

An analysis of the selected national and international publications (Chart 3) gave rise to two main themes of discussion: The Internationalization of the Red Cross and Nurses, and The Early Days of the Red Cross in Brazil and the Organization of War Nursing.

**Chart 3:** Thematic classification of news articles from the Jornal do Commercio and Gazeta de Notícias (1875–1907) – Ribeirão Preto, SP, 2025.

Newspaper	Classification	
	International	National
Jornal do Commercio	16	5
Gazeta de Notícias	11	5
Total	27	10

## DISCUSSION

### The Internationalization of the Red Cross and Nurses

The focus on the international context is set against the backdrop of armed conflicts. Throughout the second half of the 19th century and the early 20th century, the Red Cross expanded its operations, solidifying its mission as an international organization dedicated to aiding the wounded, both in times of war and during disasters.

The creation of the Red Cross, which resulted from Henri Dunant’s experience at the Battle of Solferino, transcended European borders, accompanying the outbreak of conflicts and humanitarian missions across different continents.

News reports in newspapers have contributed to shaping the image of the nursing profession throughout history and have also served various sectors in accordance with economic, social, and political interests over time^(19)^.

In this regard, as a potential avenue for studies on the promotion of professional identity and image, the importance of research in the history of nursing and its impact on the development of professionals capable of critically evaluating their own practice is particularly noteworthy^(20)^.

In this study, the newspapers analyzed highlighted the role of the International Red Cross in armed conflicts, namely: the Russo-Austrian War (1875); the Abyssinian Campaign (1896); the Eastern Revolution in Uruguay (1897); the Boer Wars (1899–1902); and the Russo-Japanese War (1904–1905).

Between 1875 and 1905, both newspapers reported on conflicts involving Russia, citing the Russian, French, and Japanese Red Cross. These include reports on field hospitals, the creation of nursing courses, the provision of care, the delivery of supplies, ambulances, war reports, accounts of the Japanese monarchy’s presence in the conflict, and the recovery of the dead and wounded/sick soldiers (Jornal do Commercio, April 5, 1875, p. 3; 02/03/1882, p. 2 and 12/18/1895, p. 2; 11/06/1899; 02/24/1904, p. 2; 07/07/1904, p. 2; 09/15/1904, p. 2; February 22, 1905, and the newspaper Gazeta de Notícias (December 18, 1895, p. 2; May 4, 1896, p. 3; April 24, 1906, p. 2).

It is worth noting that between 1875 and 1905, Russia was involved in several military conflicts, such as the Russo-Turkish War (1877–1878), which resulted in territorial gains in the Balkans as well as tensions with European powers, and the Russo-Japanese War (1904–1905), which stemmed from a defeat that exposed the fragility of the tsarist regime, contributing to the Russian Revolution of 1905.

Clara Barton, founder of the American Red Cross (1881), was featured in several news stories in both newspapers surveyed for her dedication to Cubans in their time of need.

The first report appeared on January 2, 1898, noting that she had been sent to Cuba to provide relief to peasants suffering from food shortages, as well as other reports with similar content. Months later, the book *Red Cross in the Pearl Wars* by Miss Clara Barton—president of the American Red Cross—was published in the United States (Gazeta de Notícias newspaper, January 2, 1898, p. 1; February 6, 1898, p. 1; April 27, 1898, p. 1).

Three years after the reports published in the Gazeta de Notícias, the Jornal do Commercio reported on Clara Barton’s presence on the island of Cuba to aid the local population with a grant of ten thousand dollars (Jornal do Commercio, February 5, 1901, pp. 1–8).

We recall that in 1898, Cuba fought for independence from Spain. The rebellion involved Cuban civilians. This was due to a lack of sanitation, medical supplies, and food, as well as overcrowding, which led to health complications. Barton assisted those affected during the reconcentration, when they were forced to live in precarious conditions. To this end, the Red Cross, under her leadership, established distribution centers and hospitals, providing supplies and healthcare assistance^(21)^.

Between 1900 and 1901, the Gazeta de Notícias published two news articles dedicated to the Boers. The term is of Dutch origin and, roughly translated into Portuguese, refers to the descendants of Dutch settlers or even farmers, representing those who were engaged in agriculture and livestock farming.

In this context, the Italian Red Cross sent 250 healthcare professionals—doctors and nurses—to China for a health campaign titled “Boers – The Red Cross” (Gazeta de Notícias, July 12, 1900, p. 1), and a year later, in a story titled “The Boers in Portugal”: Dutch nurses received a tribute from the Red Cross when they appeared in traditional dress, in recognition of the care they provided during the Boer War, when the dispute over control of the rich gold and diamond deposits unfolded, involving geopolitics (Gazeta de Notícias, April 18, 1901, p. 2).

The Red Cross’s presence in the locations mentioned was linked to specific storylines. On the other hand, other news items of a general nature, such as the journalistic articles “Women and War,” which report on the need for nurses in society (Jornal do Commercio, May 18, 1901, pp. 1-2); “The Religious Crisis in France,” referring to the training of nurses for times of peace and war (Jornal do Commercio, June 24, 1907, p. 2); and the article titled “England,” which referred to the creation of a faculty for nursing education (Gazeta de Notícias, June 14, 1907, p. 2). The need for women was pressing in order to train them for nursing.

Over the decades, the International Red Cross and, later, the Brazilian Red Cross knew how to use newspaper articles not only to publicize their humanitarian work but also to attract those interested in funding the institution and to recruit women for their courses^(20,22)^.

At the national level, this journalistic discourse played a public role by reporting on nurses’ work in committees, emergency situations, recruitment, and volunteer efforts, which helped to solidify the role of nurses and the importance of the Red Cross.

As an impact, it is worth noting that between World War I and World War II, 100,000 nurses were recruited into the U.S. Army and Navy, 15,000 cared for those affected by the so-called Spanish Flu in 1918, organized blood drives, and organized public and home health care^(23)^.

These characteristics appear to have persisted thereafter, as demonstrated by a study that analyzed the image of nurses using the magazine *Cruz Vermelha Brasileira* from 1942 to 1945, resulting in the portrayal of nurses as heroines, patriots, and caring angels, with the aim of fostering mass support for the war^(20)^.

### The Early Days of the Red Cross in Brazil and the Organization of Military Nursing

In the 19th century, the idea of a future Brazilian Red Cross was circulating among the country’s elite. This can be confirmed, for example, by Joaquim Nabuco’s account of the fair held at the Cassino Fluminense in Rio de Janeiro in 1893, titled “In Support of the Wounded of the Civil War in Rio Grande do Sul,” organized by the Central Red Cross Commission. At the end of his speech, he states that:

This provides insight into the concept of the Red Cross in Brazil at a time when it was not officially recognized, but we understand that the application of certain institutional principles served to inspire humanitarian action.

News about the conflict in Rio Grande do Sul—the Federalist Revolution (1893–1895)—was exploited in the publication of newspapers for readers’ consumption, which aligns with the circulation of the idea of the Red Cross, when viewed alongside headlines related to war nursing, namely: “For our suffering brothers” (Jornal do Commercio, May 21, 1893, p. 3), another with the same title (Jornal do Commercio, June 6, 1893, p. 1), “The Wounded” (Gazeta de Notícias, May 30, 1893, p. 2), “Wounded” (Gazeta de Notícias, June 6, 1893, p. 2), with the same title (Gazeta de Notícias, July 21, 1893, p. 1).

The articles published in the Jornal do Commercio addressed the creation of committees to meet the needs for nurses, Sisters of Charity, and infrastructure for blood banks (May 21, 1893, p. 3), as well as a charity fair to benefit the wounded (June 6, 1893, p. 1).

Days later, news was published that four surgeons, 2 assistants, 2 pharmacists, and 2 nurses, as well as supplies such as: 12 boxes of bandages and surgical instruments, with ratification of Article 7 regarding healthcare professionals and religious figures, who remained neutral during the conflict (May 28, 1893, p. 2; June 10, 1893, p. 1).

In 1897, the Jornal do Commercio reported on a campaign by Brazilians to organize a corps of nurses, doctors, medical assistants, and religious personnel residing in Rivera and Montevideo, Uruguay (March 21, 1897, p. 1; March 24, 1897, p. 1), as part of a strategy involving Brazil.

In the Gazeta de Notícias, there were three news items. The first contained a letter from Dr. Manoel de Penha Mendonça, who volunteered to work in the blood hospitals of Rio Grande do Sul, alongside his brother-in-law, Mr. Antonio Motilha, who would serve as a nurse (May 30, 1893, p. 2); the second article reports that a fair was held at the Cassino Fluminense, organized by the Central Commission to raise funds to meet the demands of caring for the wounded, with Mr. Hilário de Gouvêa volunteering to serve as a nurse (June 6, 1892, p. 2); and the third article details the approval of the staff roster to serve in three field hospitals and others in the surrounding area, staffed by healthcare personnel such as surgeons, pharmacists, nurses, medical assistants, and pharmacy managers (July 21, 1893, p. 1).

It is worth noting that Hilário de Gouveia or Gouvêa was an ophthalmologist. An article in the *Gazeta de Notícias* (June 6, 1892, p. 2) reports that he served as a nurse during the conflict. Considering this possibility led us to cross-reference the information. We found that he was put in charge of building a steam laundry at the Hospital dos Lázaros^(25)^ for his concern regarding nosocomial infections and the confirmation, as stated in his biography provided by the National Academy of Medicine, that he served as a physician during the conflict^(26)^, which leads us to believe that the news report intended to frame the issue as an environmental concern or that there was a misrepresentation, but we will leave that for another time.

The reports on the conflict in Rio Grande do Sul, as found in both newspapers, complement each other. The quermesse draws attention through its connection to the content in Joaquim Nabuco. This event is dated July 2, 1893, but must have occurred at least prior to the articles published in the Jornal do Commercio and the Gazeta de Notícias, both published on June 2, 1893. Thus, in fact, the idea of the Red Cross in Brazil was already circulating among the country’s elite.

The result was the dispatch of personnel and supplies to meet the needs of the wounded, possibly through funds raised at the fair, with the speech appealing to the public by referencing the Red Cross.

Jornal do Commercio ran two stories, and Gazeta de Notícias ran three. However, the first two were published close together and featured very similar headlines. This created a certain mental cue for readers to continue reading. Thus, it was one of the strategies to encourage readers to consume the newspaper through the continuity of the text on the events, which leads us to infer that, in addition to being referenced as an example of humanity and solidarity—which points to evidence for the creation of the society—it was only established years later (1908).

In editions from the early 20th century through 1907, the theme of the Red Cross reappears under the title “Red Cross Society,” featuring content on the biography of the intellectual mentor (Jornal do Commercio, June 8, 1901, pp. 1–8) and the newspaper Gazeta de Notícias under the title Henri Dunant, featuring Olavo Bilac’s account of his biography (Gazeta de Notícias, September 25, 1904, p. 3).

The articles reignite the flame of interest in the Red Cross in Brazil by publicizing the laudatory biography of Henri Dunant. To think along these lines is to refer to the inculcation of belonging to a particular group^(27)^.

To this end, a review of the Society’s international history in the Americas reveals that the Peruvian Red Cross was established in 1879; the United States Red Cross in 1882; the Salvadoran Red Cross in 1885; the Costa Rican Red Cross in 1885; the Argentine Red Cross in 1888; the Venezuelan Red Cross in 1895; in 1896, in Canada; in 1897, in Uruguay; and in 1903, in Chile, for a total of nine during that period^(28)^.

Linking the Red Cross to the name of Henri Dunant and to membership in an international institution meant being part of the prestige and wielding power. Until December 1908, its establishment remained a desire, even though the idea had been circulating since 1893; however, to create it, it was necessary to meet the criteria and secure the political will.

It is worth noting that the Red Cross was closely associated with care—that is, nursing—especially in the context of international warfare, including schools for training nurses. At the same time, nursing education in Brazil was taking shape. The School for Professional Nurses, now the Alfredo Pinto School of Nursing, was founded in 1890. Prior to that date, there is no record of any other educational institution having been established for the purpose of training nurses^(29)^.

It is known that between 1905 and 1906, the educational institution trained five nurses—three women and two men^(30)^. In other words, there were difficulties in training professionals due to the low literacy rate, as well as institutional problems, given that the program was housed within the National Asylum for the Insane; the next graduation took place in 1921, after which the program continued without interruption^(29,31)^.

Also during this period (1895), the Samaritano Hospital in São Paulo established the first Nightingale-style nursing school in Brazil, offering a three-year training program and placing nursing supervision in the hands of resident nurses^(32)^.

It is noted that registered nurses in Brazil, particularly in Rio de Janeiro, were rare prior to 1906, but in practice, those who provided care were referred to by the same title. At the time, the term was understood to refer to people who cared for the sick, without any formal training, as illustrated in the short story “The Nurse” (1896) by Machado de Assis^(33)^.

To consider this perspective is to refer to the article published in the newspaper Gazeta de Notícia (November 8, 1903, p. 2), titled “Life at São Sebastião Hospital.” The article describes the hospital’s location on the former Retiro Saudoso beach, at the tip of the Caju neighborhood, as a healthcare facility for those afflicted with infectious diseases, including a description of the care provided by the nurses.

So far, we have seen how the idea of the Red Cross spread in Brazil, the training of professionals in Rio de Janeiro, and where nursing was practiced. From this point on, we will discuss the need to train nurses for war and to respond to public disasters.

The Professional School of Nurses, attached to the National Asylum for the Insane, was established to train civilian and military professionals for hospitals and asylums under Decree No. 791/1890. The demand for professionalization arose from the departure of the Sisters of Charity, as mentioned earlier. Consequently, trained personnel were needed to provide institutional care.

Whereas Brazil lacked experience in international armed conflicts, with the exception of the Cisplatine War (1825–1828), when the Empire of Brazil fought against the United Provinces of the River Plate for control of the Cisplatine territory^(34)^, at that time under Uruguayan control, and the War of the Triple Alliance, known as the Paraguayan War (1864–1870). This did not mean there was a lack of border defense or resistance to invasions and battles, but these conflicts were incomparable to the major battles fought, for example, in North America and Europe.

The Professional School of Nurses focused on training for the care of the mentally ill, but without losing sight of the broader field of healthcare: hospitals. This served as a job market, given the social demand at the time. The Samaritan Hospital in São Paulo also met a social need, stemming from disparities in patient care due to religious choices^(32)^.

The major conflicts in the country did not draw attention to this field of education, despite participation in two wars. Health issues such as tuberculosis, yellow fever, and syphilis, among others, were ravaging the population.

The Red Cross’s focus was on major conflicts, and the trigger for the emergence of this concept, in terms of historical record, was the Federalist Revolution (1893–1895) in Rio Grande do Sul. However, the institution was not focused solely on conflicts, but also on disasters. This brings to mind Sun Tzu’s quote: “In peace, prepare for war; in war, prepare for peace.”^(35)^ In this context, the institution aims to apply these teachings to socio-political and public health issues, for example.

The Red Cross, in fact, placed an emphasis on care provided during conflicts. On the other hand, its principles—humanity, impartiality, neutrality, independence, voluntary service, unity, and universality—can be applied to public disasters.

War nursing is believed to have its roots in the military. This is in support of border defense and its survival training strategies and similar aspects. The scenario requires preparation, and it was in this context that the profession distinguished itself through professional reconfiguration with Florence Nightingale and her experience in the Crimean War (1853–1856), leading to various developments, notably the establishment of the School of Nursing at St. Thomas’ Hospital (1860), in London, England, due to the poor public health conditions prevailing in England at the time, which, within its limitations, stemmed from the conflict she had experienced^(36)^.

In Brazil, the formation of the Red Cross in the Americas is associated with Anna Justina Ferreira Nery during the Paraguayan War (1864–1870), as she volunteered to care for the wounded and sick during the conflict; however, this had no direct impact on nursing education in the country.

In any case, thinking about advances in care requires at least two paths to be pursued, through the lens of gender, which branch out into any situation where human beings of either gender find themselves in life-threatening situations^(37)^.

The study reveals that, prior to the formal establishment of the Red Cross in Brazil, the Brazilian press produced content that linked nursing, war, and relief efforts, creating a context of public contribution and visibility for care, and shaping a social image that highlighted the key figures in this field—namely, the people who provide care and how that care is delivered.

## CONCLUSION

The study offers new perspectives in the field of research, as it presents, within the journalistic text, evidence of the visibility of the Red Cross and the work of nurses prior to 1908, demonstrating that both the Red Cross institution and the work of nurses associated with these news stories were visible.

Beyond reporting facts, the newspaper played an important role in shaping the visibility of nurses and the Red Cross, both nationally and internationally, in journalistic texts between 1864 and 1907. On the international stage, the Red Cross was highlighted in wartime conflicts and for its role in neutrality, solidarity, and the organization of care for the wounded. On the national stage, as during the Federalist Revolution, the institution’s humanitarian principles were disseminated and adopted by local associations to benefit those in need, even before Brazil joined the International Red Cross Society, progressively expanding its presence in the social imagination through newspaper pages. This linked journalistic discourse primarily to its role in ongoing conflicts, but also in times of peace and in the training of professionals, which contributed to the recognition of both.

The field of journalism, as a space for the production of memory, raised the institution’s profile as a setting for nurses’ work—whether in hospitals or on the front lines—highlighting, in these two newspapers, values such as courage, competence, patriotism, and charity. Furthermore, the central idea emerging from this discussion may provide clues regarding the circulation of the idea to establish the Brazilian Red Cross.

The press, therefore, constituted an important space for the construction of images and discourses regarding the Red Cross and the work of nurses, which may have contributed to fostering the ideas of creating and establishing the organization in Brazil in the early 20th century, as well as the influence of the Red Cross’s image on the identity and image of nursing, given that it also became an important space for the training of nursing professionals worldwide.

This study contributes to discussions on the history of the nursing profession and to the teaching of the history of nursing by fostering debate on the press as a source and its impact on professional visibility, its role in disaster and war scenarios, and even the analysis of sources as expressions of political, ideological, and economic positions.

Among the limitations, the extensive time frame stands out, and, for this reason, the choice of two periodicals from that period. However, it opens up possibilities for studies in other newspapers and for expanding the documentary corpus and scope of discussion.

## Data Availability

The entire dataset supporting the results of this study has been made available at: https://bndigital.bn.gov.br/hemeroteca-digital.
