# Supplementary material for: The international Red Cross and nurses in the context of the Brazilian press (1864–1907)
Source: Rev Esc Enferm USP. 2026 Oct 26;60:e20250420. doi: 10.1590/1980-220X-REEUSP-2025-0420en (PMC13752869; doi:10.1590/1980-220X-REEUSP-2025-0420en)
Supplement: Translation

# Translation: Cruz Vermelha internacional e enfermeiros no contexto do jornal brasileiro (1864–1907)*

**DOI:** 10.1590/1980-220X-REEUSP-2025-0420pt

**Authors:** Adriana Saturnino Mazziero, Luciana Barizon Luchesi, Fernando Porto, Claudia Labriola, Paulo Joaquim Pina Queirós

**Affiliations:** 1 Universidade de São Paulo, Escola de Enfermagem de Ribeirão Preto, Ribeirão Preto, SP, Brasil.; 2 Universidade Federal do Estado do Rio de Janeiro, Escola de Enfermagem Alfredo Pinto, Rio de Janeiro, RJ, Brasil.; 3 Universidade de Coimbra, Escola Superior de Enfermagem, Unidade de Investigação em Ciências da Saúde: Enfermagem, Portugal.

**Keywords:** Enfermagem, História da Enfermagem, Cruz Vermelha, Publicação Periódica, Meios de Comunicação de Massa

## Abstract

**Objetivo:**

Investigar a visibilidade da Cruz Vermelha e do trabalho de enfermeiras/as no âmbito nacional e internacional no período 1864–1907.

**Método:**

Estudo no campo da História, utilizando análise documental do texto jornalístico, tendo como local de busca a Hemeroteca Digital Brasileira.

**Resultados:**

Foram identificadas 37 notícias, sendo 21 no Jornal do Commercio e 16 na Gazeta de Notícias. No âmbito internacional, a maioria das matérias abordou conflitos bélicos, destacando o papel da Cruz Vermelha em hospitais de sangue, reforçou a relevância do papel da enfermagem na guerra e a necessidade de formação profissional. Em âmbito nacional, destaque para a Revolução Federalista (1893–1895), com a organização de comissões de socorros e envio de voluntários, como enfermeiros, médicos e religiosos, com realização de práticas humanitárias durante os conflitos internos e a construção de identidade ligada aos princípios internacionais da Cruz Vermelha.

**Conclusão:**

A imprensa brasileira de 1864 a 1907 colaborou na divulgação da Cruz Vermelha e na valorização da enfermagem de guerra.

## INTRODUÇÃO

A Cruz Vermelha Brasileira foi criada em 1908, apesar da ideia circular desde o século XIX. Ela é descrita em diversos clássicos da história no campo da saúde e da enfermagem^(1–4)^. O contexto anterior ao surgimento da instituição no Brasil ainda é pouco explorado. A assertiva se deve às produções intelectuais em artigos, livros, dissertações e teses que são inúmeras, mas que partem basicamente de dois a três anos antes da criação, com algumas exceções^(5)^.

Foi a partir dessa perspectiva que partimos para a investigação do que ocorria no mundo sobre as ações da Cruz Vermelha. Para tanto, fomos em busca das fontes jornalísticas, pois elas são repletas de anúncios e notícias que representam aspectos dos vestígios e indícios deixados pelo passado para o presente como documentação para a construção da narrativa histórica^(6)^. Pensar nessa lógica é uma das possibilidades de entender o passado, mesmo que de forma verossímil, pois ele ficou lá^(7)^.

É fato que a Cruz Vermelha Internacional foi criada em 1863, em Genebra (Suíça), e que um de seus precursores foi Henry Dunant. Ele vivenciou a Batalha de Solferino, considerada uma das mais sangrentas e decisivas da invasão do Piemonte-Sardenha (1859), com a vitória das tropas francesas de Napoleão III contra as forças militares austríacas. Isso deu origem à obra Memórias de Solferino, inclusive com ajustes no título, com diversas reedições. Logo, ocorreu sensibilização para a criação da sociedade que atendesse aos feridos e necessitados nos conflitos bélicos^(8)^.

A proposta da Cruz Vermelha proliferou pelo mundo, e voluntários/as preparados/as eram necessários/as para agirem e atuarem nos campos de batalha, bem como em momentos de calamidade. Isto nos faz remeter à atuação da enfermagem nos campos de batalha, como na Guerra da Crimeia (1853–1856), com Florence Nightingale, e na Guerra da Tríplice Aliança, com Anna Justina Ferreira Nery (1864–1870) – voluntárias, ao oferecerem destaque e visibilidade à profissão desde o século XIX^(9)^.

Na esteira dos acontecimentos e argumentos apresentados, o presente estudo teve por objetivo investigar a visibilidade da instituição Cruz Vermelha e do/a enfermeiro/a no texto jornalístico brasileiro, que antecedeu a criação da Cruz Vermelha Brasileira.

## MÉTODO

### Tipo ou Desenho do Estudo

Estudo histórico, na dimensão da História Social, com uso da análise documental. Para tanto, argumentamos pela aplicação do método com base na operação historiográfica^(10)^, quando ocorre combinação de discursos e contornos contextuais para a produção textual articulada às fontes jornalísticas. Estes apresentam-se como vestígios do passado, por representarem certa mentalidade adotada à época para a investigação^(11)^.

A delimitação temporal adotada abrange o período de criação, em 1864, da Cruz Vermelha Internacional (CVI) até 1907, que antecede o ano de criação da Cruz Vermelha Brasileira (CVB).

### Critérios de Seleção

As buscas foram realizadas nos jornais Jornal do Commercio e Gazeta de Notícias, cuja escolha foi arbitrária. Contudo, destacamos que eles possuíam impacto na comercialização, inclusive, o Jornal do Commercio era comparado a New Times no Brasil. Isto se deve às inovações apresentadas^(12)^, bem como por serem documentos históricos com relevância nas pesquisas da História Geral e da Enfermagem^(13,14)^.

### Coleta de Dados

A busca das informações ocorreu na Hemeroteca Digital Brasileira, da Biblioteca Nacional, tendo como critérios adotados: inclusão com o termo enfermeiro/a somado à expressão Cruz Vermelha, dentro da delimitação temporal, sendo excluídas as propagandas, publicidades, caricaturas, e outros similares.

### Análise e Tratamento dos Dados

Para a organização dos dados, aplicou-se uma matriz de análise com as seguintes variáveis: título do periódico, linha editorial, preço por exemplar, endereço do periódico, responsáveis pelo jornal, título da matéria, data de veiculação da notícia, página de publicação, autoria da matéria, síntese da notícia, nome de pessoas citadas, local do contexto da notícia (país, estado, cidade ou similar).

As notícias foram transcritas integralmente utilizando-se a grafia original e seu conteúdo, mediante análise dos dados, foram trianguladas entre si, em busca de eixos temáticos^(5)^ e posteriormente com a literatura de aderência, para a construção da narrativa histórica.

### Aspectos éticos

Os aspectos éticos e legais para a realização da pesquisa foram pautados pela Resolução n. 510/2016 e na Lei n. 9.610/1998 dos Direitos Autorais.

## RESULTADOS

Os resultados apontaram para 37 notícias nos jornais, todas com título e nenhuma apresentou imagem: Jornal do Commercio e Gazeta de Notícias. Esses apresentam elementos de texto e de contexto para a narrativa histórica.

Os resultados apresentados no Quadro 1 evidenciam que a localização dos escritórios ficava no centro da cidade do Rio de Janeiro. Sobre os valores, podemos citar que o Jornal do Commercio, comparado à Gazeta de Notícias, era mais acessível ao consumidor pelo menor valor. Para ter uma certa ideia, no período de 1903 a 1907, os operários recebiam o salário em cerca de 78$000 e com este valor pagavam o aluguel de casa no valor de 8$000 a 30$000^(15)^. Entender a diferença na comercialização dos jornais permite argumentar o quantitativo maior de matéria veiculada no Jornal do Commercio comparado à Gazeta de Notícias. Logo, pode-se explicar, na perspectiva histórica, que o Jornal do Commercio foi criado em 1828, antes da Gazeta de Notícias, em 1875. Isto implica que o primeiro periódico tinha maior tradição comercial, o que pode justificar um valor menor para o consumo e uma maior quantidade de matéria veiculada.

**Quadro 1:** Dados de identificação dos jornais (1864 a 1907) – Ribeirão Preto, SP, 2025.

Dados de identificação	Jornal do commercio	Gazeta de notícias
Endereço	Rua do Ouvidor, 61, Rio de Janeiro	Rua do Ouvidor, n. 70, Rio de Janeiro – tipografia: Rua Sete de Setembro, n. 72, Rio de Janeiro – escritório
Valor do exemplar	$30,000 (1870–1889) $36,000 (1892) $40,000 – $50,000 (1893) $60,000 (1898)	$40,000 (1870–1889) $60,000 (1891) $100,000 (1893–1907)
Responsáveis	Julio Constancio de Villeneuve (1870–1879) Typografia de J. Villeneuve & C. (1880–1889) e Propriedade de Rodrigues & C. (13/01/1891)	Não identificado (1870–1879) Araujo & Mendes (1884–1907)
Linha editorial	Conservador moderado	Neutra
Quantidade de notícias	21	16
Total de notícias	37 notícias	

Outro resultado que chamou a atenção foram os títulos atrativos nas notícias jornalísticas para o leitor, ou seja, é o que faz vender o exemplar^(16)^(Quadro 2). Nesta linha do pensamento, a citação de personalidades (políticos, militares, enfermeiros/as, médicos, dentre outros) nas matérias é a proposta de oferecer credibilidade ao que é noticiado^(17)^, articulada às estratégias de argumentação no sentido da aplicação da técnica de Atenção, Interesse, Desejo e Ação (AIDA), o que leva à formação da opinião pública, especialmente dos leitores desavisados pela força da linguagem publicitária^(16)^.

**Quadro 2:** Distribuição das matérias de jornal por fonte, data e título – Ribeirão Preto, SP, 2025.

Nome do jornal	Título da matéria
1. Jornal do Commercio, 05/04/1875, p.3	Sociedade de socorros aos soldados e doentes da armada e do exército. Pariz, 8 de março de 1875
2. Gazeta de Noticias, 03/02/1882, p.2	A agitação Niilista
3. Jornal do Commercio, 21/05/1893, p.3	Para os irmãos que sofrem
4. Gazeta de Noticias, 23/05/1893, p.2	Os Feridos
5. Jornal do Commercio, 28/05/1893, p.2	Convenção da Cruz Vermelha
6. Gazeta de Noticias, 30/05/1893, p.2	Os Feridos
7. Jornal do Commercio, 06/06/1893, p.1	Pelos irmãos que sofrem
8. Gazeta de Noticias, 06/06/1893, p.2	Feridos
9. Jornal do Commercio, 10/06/1893, p.1	Pelos irmãos que sofrem
10. Gazeta de Noticias, 21/07/1893, p.1	Os Feridos
11. Gazeta de Noticias, 18/12/1895, p.2	Berlim, 2
12. Jornal do Commercio, 04/05/1896, p.3	Italianos na Africa
13. Jornal do Commercio, 21/03/1897, p.1	Telegrammas. Montevidéu, 20 de março
14. Jornal do Commercio, 24/03/1897, p.1	A Revolução do Estado Oriental
15. Gazeta de Noticias, 02/01/1898, p.1	Washington, 1
16. Gazeta de Noticias, 06/02/1898, p.1	Washington, 5
17. Gazeta de Noticias, 27/04/1898, p.1	Washington, 5
18. Jornal do Commercio, 06/11/1899, p.1	Pariz, 5 de novembro
19. Jornal do Commercio, 21/12/1899, p.1	O Dr. Camara Pestana
20. Gazeta de Noticias, 23/12/1899, p.3	Transvaal – Londres
21. Gazeta de Noticias, 12/07/1900, p.1	Boxers – A Cruz Vermelha– Roma, 11
22. Gazeta de Noticias, 18/04/1901, p.2	Os boers em Portugal
23. Jornal do Commercio, 05/02/1901, p.1	Várias Noticias Washington, 5 de fevereiro
24. Jornal do Commercio, 08/06/1901, p.2	Sociedade da Cruz Vermelha
25. Jornal do Commercio, 18/05/1901, p.2	As mulheres e a guerra
26. Jornal do Commercio, 08/06/1901, p.2	Sociedade da Cruz Vermelha
27. Gazeta de Noticias, 08/11/1903, p.2	A vida no hospital S. Sebastião
28. Jornal do Commercio, 24/02/1904, p.1	Telegrammas – Roma, 24 de fevereiro
29. Jornal do Commercio, 28/04/1904, p.1	A guerra Russo-Japoneza
30. Jornal do Commercio, 07/07/1904, p.1	Constantinopla. 6 de julho
31. Jornal do Commercio, 15/09/1904, p.1	S. Petersburgo, 14
32. Gazeta de Noticias, 25/09/1904, p.3	Henri Dunant
33. Jornal do Commercio, 05/10/1904, p.2	Várias Noticias
34. Jornal do Commercio, 22/02/1905, p.1	Londres, 21
35. Gazeta de Noticias, 24/04/1906, p.2	Tokio. 23
36. Jornal do Commercio, 24/05/1907, p.2	A crise religiosa em França (Do nosso correspondente). Pariz, 30 de Maio de 1907
37. Gazeta de Noticias, 14/06/1907, p.2	Inglaterra – Gazeta – Londres, 13

Fonte: Hemeroteca Digital Brasileira – https://bndigital.bn.gov.br/hemeroteca-digital/.

A ausência de imagens nas matérias selecionadas é compreensível, apesar do fotojornalismo ter marcação temporal em 1842^(18)^, sendo mais comuns nos semanários, mas o que não excluía a veiculação nos jornais, apesar do investimento robusto necessário.

Mediante a análise das nacionais e internacionais selecionadas (Quadro 3), deu-se origem a dois eixos temáticos da discussão: Internacionalização da Cruz Vermelha e Enfermeiros/as e Primórdios da Cruz Vermelha no Brasil e organização da enfermagem de guerra.

**Quadro 3:** Classificação temática das notícias do Jornal do Commercio e Gazeta de Notícias (1875–1907) – Ribeirão Preto, SP, 2025.

Jornal	Classificação	
	Internacional	Nacional
Jornal do Commercio	16	5
Gazeta de Notícias	11	5
Total	27	10

## DISCUSSÃO

### Internacionalização da Cruz Vermelha e Enfermeiros/as

O destaque do contexto internacional tem como cenário de fundo os conflitos bélicos. Ao longo da segunda metade do século XIX e do início do século XX, a atuação da Cruz Vermelha se expandiu, consolidando o seu propósito como organização internacional de socorros aos feridos, tanto em tempos de guerra como em calamidades.

A criação da Cruz Vermelha, que tem sua criação como resultado da experiência de Henri Dunant na Batalha de Solferino, ultrapassou fronteiras europeias, acompanhando a eclosão de conflitos e missões humanitárias em diferentes continentes.

Notícias veiculadas em jornais contribuem para a construção da imagem do profissional de enfermagem ao longo da história e também atendem a diversos setores em função de interesses econômicos, sociais e políticos ao longo do tempo^(19)^.

Nesse sentido, enquanto possibilidade de estudos sobre a divulgação da identidade e imagem profissional, destaca-se a importância da pesquisa em História da Enfermagem e seus efeitos para a formação de profissionais críticos frente ao próprio exercício profissional^(20)^.

No presente estudo, os jornais analisados destacaram a atuação da Cruz Vermelha Internacional em conflitos bélicos, a saber: Guerra Russo-Áustria (1875); Campanha da Abissínia (1896); Revolução Oriental no Uruguai (1897); Guerras dos Boers (1899–1902) e; Guerra Russo-Japonesa (1904–1905).

No período de 1875 a 1905, ambos os jornais veicularam os conflitos com envolvimento da Rússia com citação da Cruz Vermelha da Rússia, Francesa e Japonesa. Trata-se de relatos de hospital de campanha, criação de curso para enfermeiros, prestação de cuidados, encaminhamento de insumos, ambulâncias, relatórios de guerra, registro da monarquia japonesa presente no conflito e resgate de mortos e soldados feridos/enfermos (Jornal do Commercio, 05/04/1875, p. 3; 03/02/1882, p. 2 e 18/12/1895, p. 2; 06/11/1899; 24/02/1904, p. 2; 07/07/1904, p. 2; 15/09/1904, p. 2; 22/02/1905 e Jornal Gazeta de Notícias 18/12/1895, p. 2; 04/05/1896, p. 3; 24/04/1906, p. 2).

Cabe destacar que o período de 1875 a 1905, a Rússia esteve envolvida em diversos conflitos bélicos, por exemplo, a Guerra Russo-Turca (1877–1878) resultante de ganhos territoriais nos Bálcãs, bem como tensões com as potências europeias e a Guerra Russo-Japonesa (1904–1905), oriunda da derrota ao expor a fragilidade do regime czarista, o que contribuiu para a Revolução Russa de 1905.

Clara Barton, fundadora da Cruz Vermelha Americana (1881) foi destaque em diversas notícias em ambos os jornais pesquisados por sua dedicação aos cubanos em momento de necessidade.

A primeira ocorreu em 02/01/1898, ao citar que ela foi enviada para Cuba para realizar socorro aos camponeses que passavam necessidades alimentares, bem como outras com o mesmo conteúdo e meses depois a divulgação da obra Red Cross in Pearland Wars, de Miss Clara Barton – presidente da Cruz Vermelha Americana, nos Estados Unidos (Jornal Gazeta de Notícias, 02/01/1898, p. 1; 06/02/1898, p. 1; 27/04/1898, p. 1).

Três anos depois das notícias veiculadas no jornal Gazeta de Notícias, o Jornal do Commercio veiculou a presença de Clara Barton na ilha de Cuba para o socorro dos nativos com subsídio de dez mil dólares (Jornal do Commercio, 05/02/1901, p. 1–8).

Lembramos que em 1898, Cuba lutou pela independência da Espanha. A rebelião envolveu os cubanos civis. Isso ocorreu devido à ausência de saneamento, insumos médicos e alimentos, além da superlotação, o que levou aos agravos à saúde. Barton auxiliou os afetados em prol da reconcentração, quando eles foram forçados a viver em condições precárias. Para tanto, a Cruz Vermelha, sob a liderança dela, estabeleceu centros de distribuição e hospitais ao levar suprimentos e assistência à saúde^(21)^.

No período de 1900 a 1901, a Gazeta de Notícias veiculou em suas páginas duas matérias jornalísticas dedicadas aos boers. O termo é de origem holandesa e em tradução aproximada para o português significa descendentes de colonos holandeses ou até mesmo fazendeiros com representação de aqueles que se dedicavam à agricultura e à pecuária.

Nesse sentido, a Cruz Vermelha Italiana enviou para China 250 profissionais de saúde – médicos e enfermeiros –para uma campanha sanitária, com o título “Boers – A Cruz Vermelha” (Gazeta de Notícias, 12/07/1900, p. 1) e um ano depois com a notícia, intitulada “Os boers em Portugal”: as enfermeiras holandesas recebem uma homenagem da Cruz Vermelha ao se apresentarem em trajes típicos, em virtude dos cuidados prestados na Guerra dos Boers, quando o ocorreu a disputa do controle das ricas jazidas de ouro e diamantes, envolvendo a geopolítica (Gazeta de Notícias, 18/04/1901, p. 2).

A presença da Cruz Vermelha nos lugares citados se conectou a enredos específicos. Por outro lado, outras notícias, de tema geral, como por exemplo, nas matérias jornalísticas “As mulheres e a guerra” que relatam a necessidade de enfermeiras na sociedade (Jornal do Commercio, 18/05/1901, p. 1-2); “A crise religiosa em França”, referente a formação de enfermeiras para tempos de paz e guerra (Jornal do Commercio, 24/06/1907, p. 2) e com o título “Inglaterra” a matéria se referia a criação do corpo de docentes para a formação de enfermeiras (Gazeta de Notícias, 14/06/1907, p. 2). A necessidade de mulheres se fazia premente em prol de suas formações para a enfermagem.

Ao longo de décadas, a Cruz Vermelha Internacional e posteriormente, a Cruz Vermelha Brasileira souberam utilizar o texto jornalístico não apenas para a divulgação de suas características humanitárias, mas também para atrair interessados em financiar a instituição e recrutar mulheres para seus cursos^(20,22)^.

No plano nacional, esse ambiente jornalístico discursivo desempenhou um papel público ao escrever sobre a atuação de enfermeiros em comissões, situações emergenciais, recrutamento e voluntariado, o que contribuiu para consolidar o papel do enfermeiro e a importância da instituição da Cruz Vermelha.

Como impacto pode-se mencionar que entre a Primeira e Segunda Guerras Mundiais 100.000 enfermeiros foram recrutados para o exército e marinha americanos, 15.000 atenderam acometidos pela denominada Gripe Espanhola em 1918, organizando doações de sangue, organizando cuidados de saúde pública e domiciliares^(23)^.

Essas características parecem permanecer posteriormente, como demonstra estudo que analisou a figura do enfermeiro, utilizando a revista intitulada Cruz Vermelha Brasileira, de 1942 a 1945, tendo como resultado a ideia de enfermeiras como heroínas, patrióticas e anjos cuidadores, no sentido de apoiar a adesão em massa à guerra^(20)^.

### Primórdios da Cruz Vermelha no Brasil e a organização da Enfermagem de Guerra

No século XIX, a ideia sobre a futura Cruz Vermelha Brasileira circulava entre a elite do país. Isso é possível de se afirmar mediante, por exemplo, o relato de Joaquim Nabuco na quermesse no Cassino Fluminense – Rio de Janeiro -, em 1893, intitulado “A favor dos feridos da Guerra Civil do Rio Grande do Sul” sob organização da Comissão Central Cruz Vermelha e ao final ao seu pronunciamento ele cita que:

A Cruz Vermelha surge neste momento como um symbolo nacional apropriado. É o signal de perigo que se levanta em todas as pontas da costa à approximação da borrasca. Ainda que ensopa de sangue, é sempre a cruz de Christo^(24)^.

Isso revela indícios sobre a ideia da Cruz Vermelha no Brasil, quando oficialmente não era reconhecida, mas entendemos que a prática de alguns princípios institucionais promovia a motivação da ação humanitária.

As notícias sobre o conflito no Rio de Grande do Sul – Revolução Federalista (1893–1895) – eram explorados no comércio dos periódicos para o consumo dos leitores, o que coaduna com a circulação da ideia da Cruz Vermelha, quando triangulado com os títulos com a enfermagem de guerra, a saber: “Para os irmãos que sofrem” (Jornal do Commercio, 21/5/1893, p. 3), outro com o mesmo título (Jornal do Commercio, 6/6/1893, p. 1), “Os feridos” (Gazeta de Notícias, 30/5/1893, p. 2), “Feridos” (Gazeta de Notícias, 6/6/1893, p. 2), com o mesmo título (Gazeta de Notícias, 21/7/1893, p. 1).

As matérias veiculadas no Jornal do Commercio abordaram a criação de comissões para atender as demandas de enfermeiros, Irmãs da Caridade e infraestrutura para hospitais de sangue (21/5/1893, p. 3), além de uma quermesse em prol dos feridos (6/6/1893, p. 1).

Dias depois, notícias foram publicizadas de que teriam sido enviados 4 cirurgiões, 2 auxiliares, 2 farmacêuticos e 2 enfermeiros, bem como materiais, tais como: 12 caixas com material de curativo e instrumentos cirúrgicos com ratificação do artigo 7, referente aos profissionais da saúde e religiosos, que eram neutros no período conflitante (28/05/1893, p. 2; 10/6/1893, p. 1).

Em 1897, o Jornal do Commercio noticiou a subscrição de brasileiros para organização de um corpo de enfermeiro, médico, praticantes e religiosos residentes em Rivera e Montevidéu/Uruguai (21/03/1897 p. 1; 24/03/1897, p. 1), como estratégia que envolvia o Brasil.

Já no jornal da Gazeta de Notícias as notícias foram três. A primeira com conteúdo de correspondência do Dr. Manoel de Penha Mendonça que se disponibilizou para atuar nos hospitais de sangue do Rio Grande do Sul, juntamente com o seu cunhado, o Sr. Antonio Motilha para atuar como enfermeiro (30/5/1893, p. 2); a segunda matéria relata que ocorreu uma quermesse no Cassino Fluminense, organizado pela Comissão Central para arrecadar fundos para atender as demandas de atendimento aos feridos com atuação do Sr. Hilário de Gouvêa para atuar como enfermeiro (6/6/1892, p. 2); e a terceira com conteúdo de aprovação do quadro de pessoal para servir em três hospitais montados e outros nas adjacências com pessoal de saúde, tais como: cirurgiões, farmacêuticos, enfermeiros, praticantes e encarregado de farmácia (21/7/1893, p. 1).

Cabe destacar que Hilário de Gouveia ou Gouvêa era médico oftalmologista. O registro na Gazeta de Notícias (6/6/1892, p. 2) relata que ele atuou como enfermeiro no conflito. Pensar nesta possibilidade, nos fez triangular a informação. Encontramos que ele foi encarregado da construção de uma lavanderia a vapor no Hospital dos Lázaros^(25)^ por ter a preocupação com a infecção nosocomial e a ratificação que ele foi médico com atuação no conflito em biografia oferecida pela Academia Nacional de Medicina^(26)^, o que nos leva a crer que a notícia queria dar sentido ao viés do cuidar pela sua preocupação com o ambiente ou que houve registro equivocado, mas isso deixamos para outro momento.

As notícias sobre o conflito no Rio de Grande do Sul, na leitura de ambos os jornais se complementam. A quermesse chama a atenção pela articulação com o conteúdo de Joaquim Nabuco. Esse com datação de 2/7/1893, mas que deve ter ocorrido no mínimo anterior às matérias veiculadas no Jornal do Commercio e na Gazeta de Notícias, ambas publicizadas em 2/6/1893. Logo, de fato, pensar na ideia da Cruz Vermelha no Brasil era circulante na elite do país.

O desdobramento foi o encaminhamento de pessoal e material para atender às demandas dos feridos, possivelmente, mediante verba arrecadada na quermesse com apelo do discurso ao usar a referência da Cruz Vermelha.

O Jornal do Commercio investiu em duas notícias e a Gazeta de Notícias em três. Contudo, as duas primeiras se aproximam nas datas ao marcarem títulos bem semelhantes. Isso demarcava certo gatilho mental para o leitor na continuação da leitura. Logo, era uma das estratégias para consumir o periódico pela continuidade do texto sobre os acontecimentos, o que nos leva à inferência de que, além de ela ser referenciada como exemplo de humanidade e solidariedade, o que nos aponta indícios para a criação da sociedade, ela só foi materializada anos depois (1908).

Nas edições do início do século XX até 1907, a temática da Cruz Vermelha reaparece com o título Sociedade da Cruz Vermelha com conteúdo sobre a biografia do mentor intelectual (Jornal do Commercio, 8/6/1901, p. 1–8) e o jornal Gazeta de Notícias com o título Henri Dunant com abordagem de Olavo Bilac sobre a biografia dele (Gazeta de Notícias, 25/9/1904, p. 3).

As matérias reacendem as brasas da chama sobre a Cruz Vermelha no Brasil ao divulgar a biografia de exaltação a Henri Dunant. Pensar nessa lógica é se remeter a inculcação para o pertencimento a um determinado grupo^(27)^.

Para tanto, em consulta sobre a trajetória internacional da sociedade no continente americano é possível afirmar que, em 1879, ocorreu a criação da Cruz Vermelha Peruana; em 1882, dos Estados Unidos da América; em 1885, em El Salvador; em 1885, na Costa Rica; em 1888, na Argentina; em 1895, na Venezuela; em 1896, no Canadá; em 1897, no Uruguai; e em 1903, no Chile, totalizando nove no período^(28)^.

Articular a Cruz Vermelha ao nome de Henri Dunant e ao pertencimento a uma instituição internacional, era fazer parte do prestígio e ter poder. Até o mês de dezembro de 1908, sua materialização era desejo, apesar da ideia circular desde 1893, mas para criá-la foi necessário atender aos critérios e à vontade política.

Cabe destacar que a Cruz Vermelha tinha aderência aos cuidados, ou seja, a enfermagem, especialmente de guerra no âmbito internacional, incluindo escolas para formação de enfermeiros(as). Em paralelo, a educação em Enfermagem no Brasil ia se organizando. A Escola de Profissionais de Enfermeiros e Enfermeiras, atual, Escola de Enfermagem Alfredo Pinto, foi criada em 1890. Anterior a essa data se desconhece outra instituição de ensino que tenha sido fundada com a finalidade de formar enfermeiros/as^(29)^.

Sabe-se que nos anos de 1905-1906 a instituição de ensino formou cinco enfermeiros/as – três mulheres e dois homens^(30)^. Em outras palavras, havia dificuldade na formação de profissionais em virtude da baixa taxa de alfabetizados e ao mesmo tempo problemas institucionais, considerando que o funcionamento ocorria nas dependências do Hospício Nacional de Alienados, sendo a próxima formatura em 1921, quando passou a ocorrer de forma ininterrupta^(29,31)^.

Ainda nesse período (1895), o Hospital Samaritano em São Paulo estabelece a primeira escola com modelo Nightingaleano no Brasil, com estabelecimento de um curso com três anos de formação e direção de enfermagem por enfermeiros em regime de internato^(32)^.

Observa-se que os/as enfermeiros/as no Brasil formados/as, no Rio de Janeiro, até 1906 eram raros, mas na prática quem exercia o cuidado tinha a mesma denominação. O termo para a época era entendido como pessoas que cuidavam dos enfermos/doentes, sem a concepção formativa, como pode ser articulado a representação no conto O Enfermeiro (1896), de autoria de Machado de Assis^(33)^.

Pensar nessa perspectiva é associar a notícia veiculada no jornal Gazeta de Notícias (08/11/1903, pág. 2), intitulada “A vida no Hospital São Sebastião”. O conteúdo apresenta a localização na antiga praia do Retiro Saudoso, na ponta do bairro do Caju, como instituição de saúde destinada aos acometidos por doenças infectocontagiosas com descrição dos cuidados pelos/as enfermeiros/as.

Até aqui vimos como a ideia da Cruz Vermelha circulava no Brasil, a formação de profissionais, no Rio de Janeiro, e onde ocorria a prática da enfermagem. A partir deste momento, iremos discutir a necessidade da formação de enfermeiros/as para guerra e atender às calamidades públicas.

A Escola Profissional de Enfermeiros e Enfermeiras, anexa ao Hospício Nacional de Alienados, era destinada à formação de profissionais, civis, militares para hospitais e hospícios pelo Decreto n. 791/1890. A demanda para a profissionalização teve por circunstância a saída das Irmãs da Caridade, como dito antes. Logo, era necessário pessoal capacitado para atender aos cuidados institucionais.

Considerando que o Brasil carecia de experiência em conflitos bélicos internacionais, exceto na Guerra Cisplatina (1825-1828), quando o Império do Brasil lutou contra as Províncias Unidas do Rio da Prata pelo controle do território Cisplatino^(34)^, atualmente sob domínio do Uruguai e a Guerra da Tríplice Aliança, conhecida como Guerra do Paraguai (1864-1870). Isto não significava ausência de defesa das fronteiras e combate às invasões e batalhas enfrentadas, mas sem comparação aos combates de monta, especialmente, do norte do continente americano e Europa, por exemplo.

A Escola Profissional de Enfermeiros e Enfermeiras tendia a formação para os cuidados aos alienados/as, mas sem perder de vista o campo maior da saúde, os hospitais. Esse como mercado de trabalho, considerando a demanda social ocorrida à época. O Hospital Samaritano em São Paulo também atendia uma demanda social, iniciada por diferenças de tratamento de pacientes em virtude de escolha religiosa^(32)^.

Os conflitos de monta no país não chamavam a atenção para esse campo do ensino, mediante a participação em dois conflitos bélicos. Os agravos à saúde eram a tuberculose, febre amarela, sífilis, dentre outros, que assolavam a população.

A ideia da Cruz Vermelha era direcionada aos conflitos de monta e o gatilho para a circulação da representação mental, em termos de registro, quando ocorreu a Revolução Federalista (1893-1895), no Rio Grande do Sul. Contudo, a instituição não visava apenas os conflitos, mas também as calamidades. Isto nos faz remeter a Sun Tzu ao citar que “Na paz, prepara-se para a guerra; na guerra, prepara-se para a paz”^(35)^ ao articular o dito, a instituição busca é inserir os ensinamentos para as desordens sociopolíticas e sanitárias, por exemplo.

A Cruz Vermelha, de fato, dava ênfase aos cuidados prestados para os conflitos. Por outro lado, por meio de seus princípios – humanidade, imparcialidade, neutralidade, independência, voluntariado, unidade e universalidade – podem ser adotados nas calamidades públicas.

A enfermagem de guerra acredita-se estar pautada no meio militar. Essa é em prol da defesa das fronteiras e suas estratégias de treinamento de sobrevivência e aspectos similares. O cenário requer preparo e foi nela que a profissão se destacou para reconfiguração profissional com Florence Nightingale e sua experiência na Guerra da Crimeia (1853-1856) com diversos desdobramentos com destaque para a criação da Escola de Enfermagem no Hospital St. Thomas (1860), em Londres/Inglaterra, em virtude da saúde pública insalubre que ocorria Inglaterra, o que se remetia, dentro de suas limitações, ao conflito por ela vivenciada^(36)^.

No Brasil, temos a formação da Cruz Vermelha nas Américas com referência a Anna Justina Ferreira Nery, na Guerra do Paraguai (1864-1870), por ter sido voluntária para a prestação de cuidados aos feridos e doentes no conflito, mas sem efeito direto para o ensino no país.

Seja como for, pensar nos avanços dos cuidados requer ao menos dois caminhos a serem percorridos, por meio do gênero, que se ramificam ou qualquer situação em que o ser humano se encontre em risco de vida exercida por ambos os gêneros^(37)^.

O estudo traz como novidade que antes da formalização da Cruz Vermelha no Brasil, a imprensa brasileira produzia conteúdos que se articulavam entre si, enfermagem, guerra e socorro, trazendo um cenário de contribuição e visibilidade pública para o cuidado, construindo uma imagem social destacando os personagens que compõem esse ambiente quem são as pessoas que realizam o cuidado e como esse cuidado é feito.

## CONCLUSÃO

O estudo apresenta novas perspectivas no campo da pesquisa, uma vez que traz, no texto jornalístico, antecedentes da visibilidade da Cruz Vermelha e do trabalho do enfermeiro anteriores a 1908, demonstrando que houve visibilidade, tanto da instituição Cruz Vermelha quanto do trabalho de enfermeiros(as) associados a essas notícias.

Para além da escrita de fatos, o jornal desempenhou um papel importante na mediação da visibilidade de enfermeiros(as) e da Cruz Vermelha, tanto no âmbito nacional como internacional, no texto jornalístico entre 1864 e 1907. No cenário internacional, houve destaque para a Cruz Vermelha nos conflitos bélicos e para seu papel de neutralidade, solidariedade e organização de cuidados aos feridos. No cenário nacional, como na Revolução Federalista, os princípios humanitários da instituição foram divulgados e incorporados por associações locais em benefício dos que necessitam, mesmo antes da adesão do Brasil à Sociedade Cruz Vermelha internacional, ampliando progressivamente seu espaço no imaginário social, por meio das páginas do jornal, associando o discurso jornalístico principalmente ao seu papel nos conflitos em curso, mas também em tempos de paz e na formação de profissionais, o que contribuiu para a valorização de ambos.

O campo jornalístico, como espaço de produção de memória, promoveu a visibilidade da instituição enquanto espaço de atuação de enfermeiros(as), seja no espaço hospitalar, seja na frente de combate, destacando, nesses dois jornais, valores como coragem, competência, patriotismo e caridade. Além disso, a ideia central resultante da discussão pode apontar indícios sobre a circulação da ideia de criação da Cruz Vermelha Brasileira.

A imprensa, portanto, constituiu importante espaço de construção de imagens e discursos sobre a entidade Cruz Vermelha e atuação de enfermeiros, o que pode ter contribuído para fomentar as ideias de criação e implantação da entidade no Brasil no início do século XX e a própria influência da imagem da Cruz Vermelha na identidade e imagem da Enfermagem, visto que essa também passa a constituir importante espaço de formação de profissionais de Enfermagem no mundo.

O estudo contribui para as discussões sobre a memória da profissão e para o ensino de História da Enfermagem, ao potencializar o debate sobre a imprensa como fonte e seu impacto na visibilidade profissional, seu papel em cenários de catástrofes e guerras e mesmo a análise de fontes enquanto posicionamento político, ideológico e econômico dos mesmos.

Entre as limitações, destacam-se a extensa delimitação temporal e, por essa razão, a escolha de dois periódicos no período. Entretanto, abre possibilidades para estudos em outros jornais e ampliações do corpus documental e de discussão.

## Data Availability

Todo o conjunto de dados que dá suporte aos resultados deste estudo foi disponibilizado em: https://bndigital.bn.gov.br/hemeroteca-digital.

# Translation: La Cruz Roja internacional y los enfermeros en la prensa brasileña (1864–1907)*

**DOI:** 10.1590/1980-220X-REEUSP-2025-0420es

**Authors:** Adriana Saturnino Mazziero, Luciana Barizon Luchesi, Fernando Porto, Claudia Labriola, Paulo Joaquim Pina Queirós

**Affiliations:** 1 Universidade de São Paulo, Escola de Enfermagem de Ribeirão Preto, Ribeirão Preto, SP, Brasil.; 2 Universidade Federal do Estado do Rio de Janeiro, Escola de Enfermagem Alfredo Pinto, Rio de Janeiro, RJ, Brasil.; 3 Universidade de Coimbra, Escola Superior de Enfermagem, Unidade de Investigação em Ciências da Saúde: Enfermagem, Portugal.

**Keywords:** Enfermería, Historia de la Enfermería, Cruz Roja, Publicación Periódica, Medios de Comunicación de Masas

## Abstract

**Objetivo:**

Investigar la visibilidad de la Cruz Roja y del trabajo de las enfermeras en el ámbito nacional e internacional durante el período 1864–1907.

**Método:**

Estudio en el campo de la Historia, utilizando el análisis documental del texto periodístico, con la Hemeroteca Digital Brasileña como fuente de búsqueda.

**Resultados:**

Se identificaron 37 noticias, 21 de ellas en el Jornal do Commercio y 16 en la Gazeta de Notícias. En el ámbito internacional, la mayoría de los artículos abordaron conflictos bélicos, destacando el papel de la Cruz Roja en los hospitales de sangre, reforzando la relevancia del papel de la enfermería en la guerra y la necesidad de la formación profesional. En el ámbito nacional, cabe destacar la Revolución Federalista (1893–1895), con la organización de comisiones de socorro y el envío de voluntarios, como enfermeros, médicos y religiosos, la realización de prácticas humanitarias durante los conflictos internos y la construcción de una identidad vinculada a los principios internacionales de la Cruz Roja.

**Conclusión:**

La prensa brasileña de 1864 a 1907 colaboró en la difusión de la Cruz Roja y en la valorización de la enfermería de guerra.

## INTRODUCCIÓN

La Cruz Roja Brasileña se creó en 1908, aunque la idea circulaba desde el siglo XIX. Se describe en diversos clásicos de la historia en el campo de la salud y la enfermería^(1–4)^. El contexto anterior al surgimiento de la institución en Brasil aún está poco explorado. Esta afirmación se debe a que, si bien existen innumerables producciones intelectuales en forma de artículos, libros, disertaciones y tesis, estas parten básicamente de dos o tres años antes de la creación de la institución, con algunas excepciones^(5)^.

Partimos de esta perspectiva para investigar lo que ocurría en el mundo en relación con las acciones de la Cruz Roja. Para ello, recurrimos a las fuentes periodísticas, ya que están repletas de anuncios y noticias que representan aspectos de los vestigios e indicios que el pasado ha dejado para el presente como documentación para la construcción de la narrativa histórica^(6)^. Pensar en esta lógica es una de las posibilidades de entender el pasado, aunque sea de manera verosímil, ya que él quedó allí^(7)^.

Es un hecho que la Cruz Roja Internacional se creó en 1863, en Ginebra (Suiza), y que uno de sus precursores fue Henry Dunant. Él vivió la Batalla de Solferino, considerada una de las más sangrientas y decisivas de la invasión del Piamonte-Cerdeña (1859), con la victoria de las tropas francesas de Napoleón III contra las fuerzas militares austriacas. Esto dio origen a la obra Memorias de Solferino, incluso con ajustes en el título y varias reediciones. Poco después, surgió la idea de crear una sociedad que atendiera a los heridos y necesitados en los conflictos bélicos^(8)^.

La propuesta de la Cruz Roja se extendió por todo el mundo, y se necesitaban voluntarios y voluntarias preparados para actuar en los campos de batalla, así como en momentos de calamidad. Esto nos remite a la actuación de la enfermería en los campos de batalla, como en la Guerra de Crimea (1853-1856), conFlorence Nightingale, y en la Guerra de la Triple Alianza, con Anna Justina Ferreira Nery (1864-1870) —voluntarias que, desde el siglo XIX, dieron protagonismo y visibilidad a la profesión^(9)^.

A raíz de los acontecimientos y argumentos presentados, el presente estudio tuvo como objetivo investigar la visibilidad de la institución Cruz Roja y del personal de enfermería en el texto periodístico brasileño, que precedió a la creación de la Cruz Roja Brasileña.

## MÉTODO

### Tipo o Diseño del Estudio

Estudio histórico, en la dimensión de la Historia Social, con uso del análisis documental. Para ello, defendemos la aplicación del método basado en la operación historiográfica^(10)^, cuando se da una combinación de discursos y contornos contextuales para la producción textual articulada con las fuentes periodísticas. Estas se presentan como vestigios del pasado, al representar cierta mentalidad adoptada en la época para la investigación^(11)^.

El marco temporal adoptado abarca el período comprendido entre la creación, en 1864, de la Cruz Roja Internacional (CVI) y 1907, año anterior a la creación de la Cruz Roja Brasileña (CVB).

### Criterios de Selección

Las búsquedas se realizaron en los periódicos *Jornal do Commercio y Gazeta de Notícias,* cuya elección fue arbitraria. Sin embargo, cabe destacar que estos tenían un impacto en la comercialización; de hecho, el Jornal do Commercio era comparado con el New York Times en Brasil. Esto se debe a las innovaciones presentadas^(12)^, así como a que son documentos históricos relevantes para las investigaciones de Historia General y de Enfermería^(13,14)^.

### Recolección de Datos

La búsqueda de información se realizó en la Hemeroteca Digital Brasileña, de la Biblioteca Nacional, con los siguientes criterios: inclusión del término enfermero/a junto con la expresión Cruz Roja, dentro del marco temporal establecido, excluyendo los anuncios, publicidades, caricaturas y otros similares.

### Análisis y Tratamiento de los Datos

Para la organización de los datos, se aplicó una matriz de análisis con las siguientes variables: título de la publicación, línea editorial, precio por ejemplar, dirección de la publicación, responsables del periódico, título del artículo, fecha de publicación de la noticia, página de publicación, autoría del artículo, resumen de la noticia, nombre de las personas citadas, lugar del contexto de la noticia (país, estado, ciudad o similar).

Las noticias se transcribieron íntegramente utilizando la ortografía original y su contenido, mediante el análisis de los datos, se trianguló entre sí, en busca de ejes temáticos^(5)^ y posteriormente con la literatura de referencia, para la construcción de la narrativa histórica.

### Aspectos éticos

Los aspectos éticos y legales para la realización de la investigación se regieron por la Resolución n.º 510/2016 y la Ley n.º 9.610/1998 de Derechos de Autor.

## RESULTADOS

Los resultados señalaron 37 noticias en los periódicos, todas con título y ninguna con imagen: Jornal do Commercio y Gazeta de Notícias. Estas presentan elementos de texto y de contexto para la narrativa histórica.

Los resultados presentados en Cuadro 1 muestran que las oficinas estaban ubicadas en el centro de la ciudad de Río de Janeiro. En cuanto a los precios, cabe destacar que el Jornal do Commercio, en comparación con la Gazeta de Notícias, resultaba más accesible para el consumidor debido a su menor costo. Para tener una idea, en el período comprendido entre 1903 y 1907, los obreros recibían un salario de alrededor de 78$000 y con este valor pagaban el alquiler de la vivienda, que oscilaba entre 8$000 y 30$000^(15)^. Comprender la diferencia en la comercialización de los periódicos permite argumentar que el Jornal do Commercio publicaba una mayor cantidad de material en comparación con la Gazeta de Notícias. Por lo tanto, se puede explicar, desde una perspectiva histórica, que el Jornal do Commercio se fundó en 1828, antes que la Gazeta de Notícias, en 1875. Esto implica que el primer periódico tenía una mayor tradición comercial, lo que puede justificar un precio de venta más bajo y una mayor cantidad de contenido publicado.

**Cuadro 1:** Datos identificadores de los periódicos (1864 a 1907) – Ribeirão Preto, SP, 2025.

Datos de identificación	Jornal do commercio	Gazeta de notícias
Dirección	Rua do Ouvidor, 61, Rio de Janeiro	Rua do Ouvidor, n. 70, Rio de Janeiro – tipografia: Rua Sete de Setembro, n. 72, Rio de Janeiro – escritório
Valor del ejemplar	$30,000 (1870–1889) $36,000 (1892) $40,000 – $50,000 (1893) $60,000 (1898)	$40,000 (1870–1889) $60,000 (1891) $100,000 (1893–1907)
Responsables	Julio Constancio de Villeneuve (1870–1879) Typografia de J. Villeneuve & C. (1880–1889) e Propriedade de Rodrigues & C. (13/01/1891)	No identificado (1870–1879) Araujo & Mendes (1884–1907)
Línea editorial	Conservador moderado	Neutra
Cantidad de noticias	21	16
Total de noticias	37 notícias	

Otro resultado que llamó la atención fueron los titulares atractivos en las noticias periodísticas para el lector, es decir, lo que hace que se venda el ejemplar^(16)^ Cuadro 2. En esta línea de pensamiento, la cita de personalidades (políticos, militares, enfermeros/as, médicos, entre otros) en los artículos tiene como objetivo ofrecer credibilidad a lo que se informa^(17)^, articulada con estrategias de argumentación en el sentido de la aplicación de la técnica de Atención, Interés, Deseo y Acción (AIDA), lo que conduce a la formación de la opinión pública, especialmente entre los lectores desprevenidos ante la fuerza del lenguaje publicitario^(16)^.

**Cuadro 2:** Distribución de los artículos periodísticos por fuente, fecha y título – Ribeirão Preto, SP, 2025.

Nombre del periódico	Título en Portugués	Título en Español
1. Jornal do Commercio, 05/04/1875, p.3	Sociedade de socorros aos soldados e doentes da armada e do exército. Pariz, 8 de março de 1875	Sociedad de socorro a los soldados y enfermos de la Armada y del Ejército. París, 8 de marzo de 1875
2. Gazeta de Noticias, 03/02/1882, p.2	A agitação Niilista	La agitación nihilista
3. Jornal do Commercio, 21/05/1893, p.3	Para os irmãos que sofrem	Para los hermanos que sufren
4. Gazeta de Noticias, 23/05/1893, p.2	Os Feridos	Los heridos
5. Jornal do Commercio, 28/05/1893, p.2	Convenção da Cruz Vermelha	Convención de la Cruz Roja
6. Gazeta de Noticias, 30/05/1893, p.2	Os Feridos	Los heridos
7. Jornal do Commercio, 06/06/1893, p.1	Pelos irmãos que sofrem	Por los hermanos que sufren
8. Gazeta de Noticias, 06/06/1893, p.2	Feridos	Heridos
9. Jornal do Commercio, 10/06/1893, p.1	Pelos irmãos que sofrem	Por los hermanos que sufren
10. Gazeta de Noticias, 21/07/1893, p.1	Os Feridos	Los heridos
11. Gazeta de Noticias, 18/12/1895, p.2	Berlim, 2	Berlín, 2
12. Jornal do Commercio, 04/05/1896, p.3	Italianos na Africa	Italianos en África
13. Jornal do Commercio, 21/03/1897, p.1	Telegrammas. Montevidéu, 20 de março	Telegramas. Montevideo, 20 de marzo
14. Jornal do Commercio, 24/03/1897, p.1	A Revolução do Estado Oriental	La revolución del Estado Oriental
15. Gazeta de Noticias, 02/01/1898, p.1	Washington, 1	Washington, 1
16. Gazeta de Noticias, 06/02/1898, p.1	Washington, 5	Washington, 5
17. Gazeta de Noticias, 27/04/1898, p.1	Washington, 5	Washington, 5
18. Jornal do Commercio, 06/11/1899, p.1	Pariz, 5 de novembro	París, 5 de noviembre
19. Jornal do Commercio, 21/12/1899, p.1	O Dr. Camara Pestana	El Dr. Camara Pestana
20. Gazeta de Noticias, 23/12/1899, p.3	Transvaal – Londres	Transvaal – Londres
21. Gazeta de Noticias, 12/07/1900, p.1	Boxers – A Cruz Vermelha– Roma, 11	Boxers – La Cruz Roja – Roma, 11
22. Gazeta de Noticias, 18/04/1901, p.2	Os boers em Portugal	Los bóers en Portugal
23. Jornal do Commercio, 05/02/1901, p.1	Várias Noticias Washington, 5 de fevereiro	Varias noticias Washington, 5 de febrero
24. Jornal do Commercio, 08/06/1901, p.2	Sociedade da Cruz Vermelha	Sociedad de la Cruz Roja
25. Jornal do Commercio, 18/05/1901, p.2	As mulheres e a guerra	Las mujeres y la guerra
26. Jornal do Commercio, 08/06/1901, p.2	Sociedade da Cruz Vermelha	Sociedad de la Cruz Roja
27. Gazeta de Noticias, 08/11/1903, p.2	A vida no hospital S. Sebastião	La vida en el hospital S. Sebastián
28. Jornal do Commercio, 24/02/1904, p.1	Telegrammas – Roma, 24 de fevereiro	Telegramas – Roma, 24 de febrero
29. Jornal do Commercio, 28/04/1904, p.1	A guerra Russo-Japoneza	La guerra ruso-japonesa
30. Jornal do Commercio, 07/07/1904, p.1	Constantinopla. 6 de julho	Constantinopla. 6 de julio
31. Jornal do Commercio,15/09/1904, p.1	S. Petersburgo, 14	San Petersburgo, 14
32. Gazeta de Noticias, 25/09/1904, p.3	Henri Dunant	Henri Dunant
33. Jornal do Commercio, 05/10/1904, p.2	Várias Noticias	Varias noticias
34. Jornal do Commercio, 22/02/1905, p.1	Londres, 21	Londres, 21
35. Gazeta de Noticias, 24/04/1906, p.2	Tokio. 23	Tokio. 23
36. Jornal do Commercio, 24/05/1907, p.2	A crise religiosa em França (Do nosso correspondente). Pariz, 30 de Maio de 1907	La crisis religiosa en Francia (De nuestro corresponsal). París, 30 de mayo de 1907
37. Gazeta de Noticias, 14/06/1907, p.2	Inglaterra – Gazeta – Londres, 13	Inglaterra – Gaceta – Londres, 13

Fuente: Hemeroteca Digital Brasileira – https://bndigital.bn.gov.br/hemeroteca-digital/.

La ausencia de imágenes en los artículos seleccionados es comprensible, a pesar de que el fotoperiodismo se remonta a 1842^(18)^, siendo más común en los semanarios, lo que no excluía su publicación en los diarios, a pesar de la considerable inversión necesaria.

A través del análisis de los artículos nacionales e internacionales seleccionados (Cuadro 3), surgieron dos ejes temáticos de discusión: La internacionalización de la Cruz Roja y el personal de enfermería, y Los inicios de la Cruz Roja en Brasil y la organización de la enfermería de guerra.

**Cuadro 3:** Clasificación temática de las noticias del Jornal do Commercio y la Gazeta de Notícias (1875–1907) – Ribeirão Preto, SP, 2025.

Periódico	Clasificación	
	Internacional	Nacional
Jornal do Commercio	16	5
Gazeta de Notícias	11	5
Total	27	10

## DISCUSIÓN

### La Internacionalización de la Cruz Roja y el personal de Enfermería

El contexto internacional tiene como telón de fondo los conflictos bélicos. A lo largo de la segunda mitad del siglo XIX y principios del siglo XX, la actuación de la Cruz Roja se expandió, consolidando su propósito como organización internacional de socorro a los heridos, tanto en tiempos de guerra como en casos de catástrofes.

La creación de la Cruz Roja, que tiene su origen en la experiencia de Henri Dunant en la batalla de Solferino, traspasó las fronteras europeas, acompañando el estallido de conflictos y misiones humanitarias en diferentes continentes.

Las noticias publicadas en los periódicos contribuyen a la construcción de la imagen del profesional de enfermería a lo largo de la historia y también atienden a diversos sectores en función de intereses económicos, sociales y políticos a lo largo del tiempo^(19)^.

En este sentido, como posibilidad de estudios sobre la difusión de la identidad y la imagen profesional, se destaca la importancia de la investigación en Historia de la Enfermería y sus efectos para la formación de profesionales críticos frente al propio ejercicio profesional^(20)^.

En el presente estudio, los periódicos analizados destacaron la actuación de la Cruz Roja Internacional en conflictos bélicos, a saber: Guerra Ruso-Austriaca (1875); Campaña de Abisinia (1896); Revolución Oriental en Uruguay (1897); Guerras de los Bóers (1899–1902) y; Guerra Ruso-Japonesa (1904–1905).

En el período comprendido entre 1875 y 1905, ambos periódicos informaron sobre los conflictos en los que participó Rusia, citando a la Cruz Roja de Rusia, Francia y Japón. Se trata de relatos sobre hospitales de campaña, la creación de cursos para enfermeros, la prestación de cuidados, el envío de suministros, ambulancias, informes de guerra, el registro de la presencia de la monarquía japonesa en el conflicto y el rescate de muertos y soldados heridos o enfermos (Jornal do Commercio, 05/04/1875, p. 3; 03/02/1882, p. 2 y 18/12/1895, p. 2; 06/11/1899; 24/02/1904, p. 2; 07/07/1904, p. 2; 15/09/1904, p. 2; 22/02/1905 y Gazeta de Notícias 18/12/1895, p. 2; 04/05/1896, p. 3; 24/04/1906, p. 2).

Cabe destacar que, durante el período comprendido entre 1875 y 1905, Rusia se vio envuelta en diversos conflictos bélicos, por ejemplo, la Guerra Ruso-Turca (1877–1878), resultado de las conquistas territoriales en los Balcanes, así como de las tensiones con las potencias europeas, y la Guerra Ruso-Japonesa (1904–1905), que surgió de la derrota al poner de manifiesto la fragilidad del régimen zarista, lo que contribuyó a la Revolución Rusa de 1905.

Clara Barton, fundadora de la Cruz Roja Americana (1881), fue destacada en varias noticias en ambos periódicos analizados por su dedicación a los cubanos en momentos de necesidad. La primera apareció el 2 de enero de 1898, al mencionar que fue enviada a Cuba para prestar ayuda a los campesinos que pasaban necesidades alimentarias, así como otras con el mismo contenido y, meses después, la difusión de la obra Red Cross in the Pearl Wars, de la señorita Clara Barton, presidenta de la Cruz Roja Americana, en los Estados Unidos (Periódico Gazeta de Notícias, 02/01/1898, p. 1; 06/02/1898, p. 1; 27/04/1898, p. 1).

Tres años después de las noticias publicadas en el periódico Gazeta de Notícias, el Jornal do Commercio informó de la presencia de Clara Barton en la isla de Cuba para socorrer a los nativos con una subvención de diez mil dólares (Jornal do Commercio, 05/02/1901, p. 1–8).

Recordamos que en 1898, Cuba luchó por su independencia de España. La rebelión involucró a la población civil cubana. Esto ocurrió debido a la falta de saneamiento, suministros médicos y alimentos, además del hacinamiento, lo que provocó problemas de salud. Barton ayudó a los afectados durante la reconcentración, cuando se vieron obligados a vivir en condiciones precarias. Para ello, la Cruz Roja, bajo su liderazgo, estableció centros de distribución y hospitales para llevar suministros y asistencia sanitaria^(21)^.

En el período de 1900 a 1901, la Gazeta de Notícias publicó en sus páginas dos artículos periodísticos dedicados a los *boers*. El término es de origen holandés y, en una traducción aproximada al español, significa descendientes de colonos holandeses o incluso granjeros, en referencia a aquellos que se dedicaban a la agricultura y la ganadería.

En este sentido, la Cruz Roja Italiana envió a China a 250 profesionales de la salud —médicos y enfermeros— para una campaña sanitaria, con el título «Boers – La Cruz Roja» (Gazeta de Notícias, 12/07/1900, p. 1) y, un año después, con la noticia titulada «Los boers en Portugal»: las enfermeras holandesas reciben un homenaje de la Cruz Roja al presentarse con trajes típicos, en reconocimiento a la atención prestada durante la Guerra de los Bóers, cuando se produjo la disputa por el control de los ricos yacimientos de oro y diamantes, lo que implicó la geopolítica (Gazeta de Notícias, 18/04/1901, p. 2).

La presencia de la Cruz Roja en los lugares citados se relacionó con contextos específicos. Por otro lado, otras noticias, de tema general, como por ejemplo, en los artículos periodísticos «Las mujeres y la guerra», que relatan la necesidad de enfermeras en la sociedad (Jornal do Commercio, 18/05/1901, p. 1-2);

«La crisis religiosa en Francia», referente a la formación de enfermeras para tiempos de paz y de guerra (Jornal do Commercio, 24/06/1907, p. 2) y, con el título «Inglaterra», el artículo se refería a la creación del cuerpo docente para la formación de enfermeras (Gazeta de Notícias, 14/06/1907, p. 2). La necesidad de mujeres se hacía apremiante en pro de su formación en enfermería.

A lo largo de décadas, la Cruz Roja Internacional y, posteriormente, la Cruz Roja Brasileña supieron utilizar el texto periodístico no solo para la difusión de sus características humanitarias, sino también para atraer a interesados en financiar la institución y reclutar mujeres para sus cursos^(20,22)^.

A nivel nacional, este entorno periodístico discursivo desempeñó un papel público al escribir sobre la actuación de los enfermeros en comisiones, situaciones de emergencia, reclutamiento y voluntariado, lo que contribuyó a consolidar el papel del enfermero y la importancia de la institución de la Cruz Roja.

Como impacto, cabe mencionar que entre la Primera y la Segunda Guerra Mundial se reclutaron 100 000 enfermeros para el ejército y la marina estadounidenses, 15 000 atendieron a afectados por la denominada Gripe Española en 1918, organizando donaciones de sangre, organizando la atención de salud pública y domiciliaria^(23)^.

Estas características parecen haber perdurado posteriormente, como lo demuestra un estudio que analizó la figura de la enfermera, utilizando la revista titulada Cruz Vermelha Brasileira, de 1942 a 1945, lo que dio como resultado la idea de las enfermeras como heroínas, patriotas y ángeles cuidadores, con el fin de apoyar la adhesión masiva a la guerra^(20)^.

### Los inicios de la Cruz Roja en Brasil y la organización de la Enfermería de Guerra

En el siglo XIX, la idea sobre la futura Cruz Roja Brasileña circulaba entre la élite del país. Esto se puede afirmar, por ejemplo, a partir del relato de Joaquim Nabuco en la fiesta benéfica en el Cassino Fluminense —Río de Janeiro—, en 1893, titulada «A favor de los heridos de la Guerra Civil de Río Grande del Sur», organizada por la Comisión Central de la Cruz Roja, y al final de su discurso cita que:

La Cruz Roja surge en este momento como un símbolo nacional apropiado. Es la señal de peligro que se alza en todos los extremos de la costa ante la aproximación de la tormenta. Aunque empapada de sangre, es siempre la cruz de Cristo^(24)^.

Esto revela indicios sobre la idea de la Cruz Roja en Brasil, cuando aún no era reconocida oficialmente, pero entendemos que la práctica de algunos principios institucionales promovía la motivación de la acción humanitaria.

Las noticias sobre el conflicto en Río Grande del Sur —la Revolución Federalista (1893-1895)— eran explotadas en el mercado de los periódicos para el consumo de los lectores, lo que concuerda con la difusión de la idea de la Cruz Roja, cuando se relaciona con los titulares sobre la enfermería de guerra, a saber: «Para los hermanos que sufren» (Jornal do Commercio, 21/5/1893, p. 3), otro con el mismo título (Jornal do Commercio, 6/6/1893, p. 1), «Los heridos» (Gazeta de Notícias, 30/5/1893, p. 2), «Heridos» (Gazeta de Notícias, 6/6/1893, p. 2), con el mismo título (Gazeta de Notícias, 21/7/1893, p. 1).

Los artículos publicados en el Jornal do Commercio abordaron la creación de comisiones para atender las demandas de enfermeros, Hermanas de la Caridad e infraestructura para hospitales de sangre (21/5/1893, p. 3), además de una feria benéfica en favor de los heridos (6/6/1893, p. 1).

Días después, se publicaron noticias de que se habrían enviado 4 cirujanos, 2 auxiliares, 2 farmacéuticos y 2 enfermeros, así como materiales, tales como: 12 cajas con material de curación e instrumentos quirúrgicos, con ratificación del artículo 7, referente a los profesionales de la salud y religiosos, que se mantuvieron neutrales durante el período de conflicto (28/05/1893, p. 2; 10/6/1893, p. 1).

En 1897, el Jornal do Commercio informó sobre la suscripción de brasileños para la organización de un cuerpo de enfermeros, médicos, practicantes y religiosos residentes en Rivera y Montevideo/Uruguay (21/03/1897 p. 1; 24/03/1897, p. 1), como estrategia que involucraba a Brasil.

En el periódico Gazeta de Notícias, por su parte, se publicaron tres noticias. La primera contenía una carta del Dr. Manoel de Penha Mendonça, quien se ofreció para trabajar en los hospitales de sangre de Rio Grande do Sul, junto con su cuñado, el Sr. Antonio Motilha, quien se ofrecería como enfermero (30/5/1893, p. 2); la segunda noticia relata que se celebró una fiesta benéfica en el Cassino Fluminense, organizada por la Comisión Central para recaudar fondos destinados a atender las necesidades de los heridos, con la participación del Sr. Hilário de Gouvêa como enfermero (6/6/1892, p. 2); y el tercero contiene la aprobación de la plantilla de personal para prestar servicio en tres hospitales de campaña y otros en las inmediaciones con personal sanitario, tales como: cirujanos, farmacéuticos, enfermeros, practicantes y encargados de farmacia (21/7/1893, p. 1).

Cabe destacar que Hilário de Gouveia o Gouvêa era médico oftalmólogo. El registro en la Gazeta de Notícias (6/6/1892, p. 2) relata que ejerció como enfermero en el conflicto. Pensar en esta posibilidad nos llevó a contrastar la información. Descubrimos que estuvo a cargo de la construcción de una lavandería a vapor en el Hospital dos Lázaros^(25)^ debido a su preocupación por las infecciones nosocomiales, y la confirmación de que fue médico en activo durante el conflicto en la biografía proporcionada por la Academia Nacional de Medicina^(26)^, lo que nos lleva a creer que la noticia quería dar sentido al sesgo del cuidado por su preocupación por el medio ambiente o que hubo un registro erróneo, pero eso lo dejamos para otro momento.

Las noticias sobre el conflicto en Rio Grande do Sul, en la lectura de ambos periódicos, se complementan. La quermesse llama la atención por su relación con el contenido de Joaquim Nabuco. Este tiene fecha del 2/7/1893, pero debe haber ocurrido como mínimo antes de los artículos publicados en el Jornal do Commercio y en la Gazeta de Notícias, ambos publicados el 2/6/1893. Por lo tanto, de hecho, la idea de la Cruz Roja en Brasil circulaba entre la élite del país.

El resultado fue el envío de personal y material para atender las necesidades de los heridos, posiblemente mediante fondos recaudados en la feria con un discurso que hacía referencia a la Cruz Roja.

El Jornal do Commercio publicó dos noticias y la Gazeta de Notícias, tres. Sin embargo, las dos primeras se acercan en las fechas al presentar títulos muy similares. Esto marcaba un cierto estímulo mental para que el lector continuara la lectura. Por lo tanto, era una de las estrategias para consumir el periódico a través de la continuidad del texto sobre los acontecimientos, lo que nos lleva a la inferencia de que, además de ser referenciada como ejemplo de humanidad y solidaridad —lo que nos da indicios de la creación de la sociedad—, esta solo se materializó años después (1908).

En las ediciones de principios del siglo XX hasta 1907, la temática de la Cruz Roja reaparece con el título Sociedad de la Cruz Roja, con contenido sobre la biografía del mentor intelectual (Jornal do Commercio, 8/6/1901, p. 1–8) y el periódico Gazeta de Notícias con el título Henri Dunant, con un enfoque de Olavo Bilac sobre su biografía (Gazeta de Notícias, 25/9/1904, p. 3).

Los artículos reavivan las brasas de la llama sobre la Cruz Roja en Brasil al difundir la biografía de exaltación a Henri Dunant. Pensar en esta lógica es remitirse a la inculcación de la pertenencia a un determinado grupo^(27)^.

Para ello, al consultar la trayectoria internacional de la sociedad en el continente americano, es posible afirmar que, en 1879, se creó la Cruz Roja Peruana; en 1882, la de los Estados Unidos de América; en 1885, la de El Salvador; en 1885, la de Costa Rica; en 1888, la de Argentina; en 1895, la de Venezuela; en 1896, en Canadá; en 1897, en Uruguay; y en 1903, en Chile, sumando un total de nueve en ese período^(28)^.

Vincular a la Cruz Roja con el nombre de Henri Dunant y con la pertenencia a una institución internacional era sinónimo de prestigio y poder. Hasta diciembre de 1908, su materialización era un deseo, a pesar de que la idea circulaba desde 1893, pero para crearla fue necesario cumplir con los criterios y contar con la voluntad política.

Cabe destacar que la Cruz Roja se dedicaba a la atención sanitaria, es decir, a la enfermería, especialmente de guerra en el ámbito internacional, incluyendo escuelas para la formación de enfermeros y enfermeras. Paralelamente, la educación en enfermería en Brasil se iba organizando. La Escuela de Profesionales de Enfermería, actual Escuela de Enfermería Alfredo Pinto, fue creada en 1890. No se conoce ninguna otra institución educativa anterior a esa fecha que haya sido fundada con el propósito de formar enfermeros^(29)^.

Se sabe que en los años 1905-1906 la institución educativa formó a cinco enfermeros: tres mujeres y dos hombres^(30)^. En otras palabras, existía dificultad en la formación de profesionales debido a la baja tasa de alfabetización y, al mismo tiempo, a problemas institucionales, considerando que el funcionamiento se llevaba a cabo en las instalaciones del Hospicio Nacional de Alienados, siendo la siguiente graduación en 1921, cuando comenzó a ocurrir de manera ininterrumpida^(29,31)^.

Aún en ese período (1895), el Hospital Samaritano de São Paulo estableció la primera escuela con modelo nightingaleano en Brasil, con la creación de un curso de tres años de formación y la dirección de enfermería a cargo de enfermeros en régimen de internado^(32)^.

Se observa que los enfermeros y enfermeras formados en Brasil, en Río de Janeiro, hasta 1906 eran escasos, pero en la práctica quienes ejercían el cuidado tenían la misma denominación. El término se entendía en aquella época como personas que cuidaban a los enfermos, sin la concepción formativa, tal como se puede articular la representación en el cuento *El enfermero* (1896), de autoría de Machado de Assis^(33)^.

Pensar en esta perspectiva es asociar la noticia publicada en el periódico Gazeta de Notícias (08/11/1903, pág. 2), titulada «La vida en el Hospital São Sebastião». El contenido presenta la ubicación en la antigua playa del Retiro Saudoso, en el extremo del barrio de Caju, como una institución de salud destinada a los afectados por enfermedades infecciosas, con una descripción de los cuidados prestados por los enfermeros y enfermeras.

Hasta aquí hemos visto cómo circulaba la idea de la Cruz Roja en Brasil, la formación de profesionales en Río de Janeiro y dónde se ejercía la enfermería. A partir de este momento, discutiremos la necesidad de formar enfermeros y enfermeras para la guerra y para atender las calamidades públicas.

La Escuela Profesional de Enfermeros y Enfermeras, anexa al Hospicio Nacional de Alienados, estaba destinada a la formación de profesionales, civiles y militares, para hospitales y hospicios, según el Decreto n.º 791/1890. La demanda de profesionalización se debió a la salida de las Hermanas de la Caridad, como se mencionó anteriormente. Por lo tanto, se necesitaba personal capacitado para atender los cuidados institucionales.

Teniendo en cuenta que Brasil carecía de experiencia en conflictos bélicos internacionales, salvo en la Guerra Cisplatina (1825-1828), cuando el Imperio de Brasil luchó contra las Provincias Unidas del Río de la Plata por el control del territorio cisplatino^(34)^, actualmente bajo dominio de Uruguay, y la Guerra de la Triple Alianza, conocida como Guerra del Paraguay (1864-1870). Esto no significaba una ausencia de defensa de las fronteras y de lucha contra las invasiones y batallas enfrentadas, pero sin comparación con los combates a gran escala, especialmente en el norte del continente americano y en Europa, por ejemplo.

La Escuela Profesional de Enfermeros y Enfermeras se orientaba a la formación para el cuidado de personas con enfermedades mentales, pero sin perder de vista el ámbito más amplio de la salud: los hospitales. Este era el mercado laboral, considerando la demanda social existente en la época. El Hospital Samaritano de São Paulo también atendía una demanda social, surgida por las diferencias en el tratamiento de los pacientes en virtud de la elección religiosa^(32)^.

Los conflictos de gran envergadura en el país no llamaban la atención sobre este campo de la enseñanza, a pesar de la participación en dos conflictos bélicos. Las enfermedades que asolaban a la población eran la tuberculosis, la fiebre amarilla y la sífilis, entre otras.

La idea de la Cruz Roja se orientaba hacia los conflictos de gran envergadura y el detonante para la circulación de la representación mental, en términos de registro, se produjo cuando tuvo lugar la Revolución Federalista (1893-1895) en Rio Grande do Sul. Sin embargo, la institución no se enfocaba únicamente en los conflictos, sino también en las calamidades. Esto nos remite a *Sun Tzu* al citar que «En la paz, prepárate para la guerra; en la guerra, prepárate para la paz»^(35)^; al articular este dicho, la institución busca aplicar las enseñanzas a los desórdenes sociopolíticos y sanitarios, por ejemplo.

La Cruz Roja, de hecho, ponía énfasis en la atención prestada durante los conflictos. Por otro lado, a través de sus principios —humanidad, imparcialidad, neutralidad, independencia, voluntariado, unidad y universalidad— pueden aplicarse en las catástrofes públicas.

Se cree que la enfermería de guerra se basa en el ámbito militar. Esto es en pro de la defensa de las fronteras y sus estrategias de entrenamiento de supervivencia y aspectos similares. El escenario requiere preparación y fue en él donde la profesión se destacó para la reconfiguración profesional con Florence Nightingale y su experiencia en la Guerra de Crimea (1853-1856), con diversas ramificaciones, entre las que destaca la creación de la Escuela de Enfermería en el Hospital St. Thomas (1860), en Londres, Inglaterra, debido a las precarias condiciones de salud pública que se vivían en Inglaterra, lo cual se relacionaba, dentro de sus limitaciones, con el conflicto que ella había vivido^(36)^.

En Brasil, tenemos la formación de la Cruz Roja en las Américas con referencia a Anna Justina Ferreira Nery, en la Guerra del Paraguay (1864-1870), por haber sido voluntaria para la prestación de cuidados a los heridos y enfermos en el conflicto, pero sin efecto directo para la enseñanza en el país.

Sea como fuere, pensar en los avances de la atención requiere al menos dos caminos a recorrer, a través del género, que se ramifican o cualquier situación en la que el ser humano se encuentre en riesgo de vida ejercida por ambos géneros^(37)^.

El estudio aporta como novedad que, antes de la formalización de la Cruz Roja en Brasil, la prensa brasileña producía contenidos que se articulaban entre sí —enfermería, guerra y socorro—, lo que generaba un escenario de contribución y visibilidad pública para la atención, construyendo una imagen social que destacaba a los personajes que componen ese entorno: quiénes son las personas que prestan la atención y cómo se lleva a cabo dicha atención.

## CONCLUSIÓN

El estudio presenta nuevas perspectivas en el campo de la investigación, ya que aporta, en el texto periodístico, antecedentes de la visibilidad de la Cruz Roja y del trabajo de los enfermeros anteriores a 1908, demostrando que hubo visibilidad, tanto de la institución Cruz Roja como del trabajo de los enfermeros y enfermeras asociados a estas noticias.

Más allá de la simple exposición de hechos, el periódico desempeñó un papel importante en la mediación de la visibilidad de los enfermeros y enfermeras y de la Cruz Roja, tanto en el ámbito nacional como internacional, en el texto periodístico entre 1864 y 1907. En el escenario internacional, se destacó a la Cruz Roja en los conflictos bélicos y su papel de neutralidad, solidaridad y organización de la atención a los heridos. En el ámbito nacional, como en la Revolución Federalista, los principios humanitarios de la institución fueron difundidos e incorporados por asociaciones locales en beneficio de los necesitados, incluso antes de la adhesión de Brasil a la Sociedad Internacional de la Cruz Roja, ampliando progresivamente su espacio en el imaginario social a través de las páginas del periódico, asociando el discurso periodístico principalmente a su papel en los conflictos en curso, pero también en tiempos de paz y en la formación de profesionales, lo que contribuyó a la valorización de ambos.

El ámbito periodístico, como espacio de producción de memoria, promovió la visibilidad de la institución como espacio de actuación de enfermeros y enfermeras, ya sea en el ámbito hospitalario o en el frente de combate, destacando, en estos dos periódicos, valores como el coraje, la competencia, el patriotismo y la caridad. Además, la idea central resultante de la discusión puede apuntar indicios sobre la circulación de la idea de la creación de la Cruz Roja Brasileña.

La prensa, por lo tanto, constituyó un importante espacio para la construcción de imágenes y discursos sobre la entidad Cruz Roja y la actuación de los enfermeros, lo que pudo haber contribuido a fomentar las ideas de creación e implantación de la entidad en Brasil a principios del siglo XX y la propia influencia de la imagen de la Cruz Roja en la identidad y la imagen de la enfermería, dado que esta también pasa a constituir un importante espacio de formación de profesionales de la enfermería en el mundo.

El estudio contribuye a los debates sobre la memoria de la profesión y a la enseñanza de la Historia de la Enfermería, al potenciar el debate sobre la prensa como fuente y su impacto en la visibilidad profesional, su papel en escenarios de catástrofes y guerras, e incluso el análisis de las fuentes como posicionamiento político, ideológico y económico de las mismas.

Entre las limitaciones, se destacan la extensa delimitación temporal y, por esa razón, la elección de dos periódicos en el período. Sin embargo, abre posibilidades para estudios en otros periódicos y ampliaciones del corpus documental y de discusión.

## Data Availability

Todo el conjunto de datos en el que se basan los resultados de este estudio se ha publicado em: https://bndigital.bn.gov.br/hemeroteca-digital.
